# Promoting and maintaining physical activity in the transition to retirement: a systematic review of interventions for adults around retirement age

**DOI:** 10.1186/s12966-016-0336-3

**Published:** 2016-02-01

**Authors:** S. Baxter, M. Johnson, N. Payne, H. Buckley-Woods, L. Blank, E. Hock, A. Daley, A. Taylor, T. Pavey, G. Mountain, E. Goyder

**Affiliations:** School of Health and Related Research, University of Sheffield, Regent Court, 30 Regent Street, Sheffield, S14DA UK; Institute of Applied Health Research, College of Medical and Dental Sciences, University of Birmingham, Birmingham, UK; Peninsula Schools of Medicine & Dentistry, Plymouth University, Plymouth, UK; School of Human Movement and Nutrition Sciences, University of Queensland, Brisbane, Australia

**Keywords:** Physical activity, Retirement, Older age, Exercise, Inequalities, Systematic review, Interventions, Programmes

## Abstract

It has been argued that transition points in life, such as the approach towards, and early years of retirement present key opportunities for interventions to improve the health of the population. Research has also highlighted inequalities in health status in the retired population and in response to interventions which should be addressed. We aimed to conduct a systematic review to synthesise international evidence on the types and effectiveness of interventions to increase physical activity among people around the time of retirement. A systematic review of literature was carried out between February 2014 and April 2015. Searches were not limited by language or location, but were restricted by date to studies published from 1990 onwards. Methods for identification of relevant studies included electronic database searching, reference list checking, and citation searching. Systematic search of the literature identified 104 papers which described study populations as being older adults. However, we found only one paper which specifically referred to their participants as being around the time of retirement. The intervention approaches for older adults encompassed: training of health care professionals; counselling and advice giving; group sessions; individual training sessions; in-home exercise programmes; in-home computer-delivered programmes; in-home telephone support; in-home diet and exercise programmes; and community-wide initiatives. The majority of papers reported some intervention effect, with evidence of positive outcomes for all types of programmes. A wide range of different measures were used to evaluate effectiveness, many were self-reported and few studies included evaluation of sedentary time. While the retirement transition is considered a significant point of life change, little research has been conducted to assess whether physical activity interventions at this time may be effective in promoting or maintaining activity, or reducing health inequalities. We were unable to find any evidence that the transition to retirement period was, or was not a significant point for intervention. Studies in older adults more generally indicated that a range of interventions might be effective for people around retirement age.

**Electronic supplementary material:**

The online version of this article (doi:10.1186/s12966-016-0336-3) contains supplementary material, which is available to authorized users.

## Background

With a growing population approaching retirement age there has been an increasing focus on how this sector of society can maintain their independence, and their mental and physical well-being. It has been recognised for some years that a large proportion of people aged over 50 are sedentary (take less than half an hour of moderate intensity physical activity a week), and few take levels of activity recommended for improving health (30 min of moderate physical activity at least five times a week such as brisk walking) [1].

It has been argued that significant points of life change (such as the approach towards, and early years of retirement) present an opportunity for health promotion activities and other interventions [1, 2]. The transition to retirement may be associated with significant alterations in lifestyle, including change in the level and types of physical activity [PA]. This period of life may therefore be a potentially important time to intervene, to maintain or promote activity in older age [2, 3, 4]. Recent research also suggests that total weekly PA levels after retirement tend to increase in older people from higher socioeconomic groups, but decrease in those from lower socioeconomic groups [1]. There is therefore potential for PA interventions to address widening health inequalities in older age.

A number of previous reviews have examined PA interventions in populations of older adults (over 50 s, over 60 s, over 65 s, 55–75 year olds or older adults generally), or associations between life changes and PA [5–16]. Three previous reviews have specifically considered retirement [1, 16, 17]. The first of these [16] examined health promotion at retirement, the second [1] examined studies which reported associations between exercise and PA in retirement, the third examined five qualitative papers relating to the retirement transition [17]. A table is available as Additional file 1 which details the focus and findings of these reviews.

Our study, in contrast to this existing work, aimed to conduct a systematic review of international evidence on the types and effectiveness of interventions to increase PA among people around the time of retirement. We aimed to examine evidence regarding optimal interventions around this point of life change. Also, we intended to examine the impact of interventions in different populations, and the potential for retirement to increase health inequalities.

## Review

A review protocol was developed prior to beginning the study, and was registered with the PROSPERO database number CRD: 42014007446.

### Identification of studies

A systematic and comprehensive search of electronic databases was undertaken in March 2014 to December 2014. A wide variety of sources were searched in the disciplines of medicine and health, social sciences, and specialist bibliographic databases. The initial search comprised terms to reflect the concept of the transition into retirement, combined with terms to reflect the concept of physical activity. This search retrieved only a limited number of relevant papers, therefore a second search was developed which used broader terms for older age. The databases searched and search strategy are available as Additional file 1.

In addition to electronic database searching, citation searching was undertaken, and searches for grey literature from the UK, together with screening of reference lists of included studies.

All citations were imported into Reference Manager (Version 12) and duplicates deleted. The database of citations was screened at title and abstract level by two members of the team.

Our target population was people during and shortly after the transition to retirement, including those not in paid employment, and those about to leave paid employment. We excluded study populations where the intervention was provided for a specific clinical condition, participants described as being elderly and frail, or with limited mobility. We included studies evaluating any intervention which aimed to increase and/or maintain levels of physical activity. We excluded interventions which were described as aiming to increase stretching/flexibility/balance, or reduce falls.

Outcomes of interest included direct measures of physical activity, indirect measures of physical activity (such as hours of gardening), and relevant social, psychological, behavioural and environmental outcomes. Experimental and observational studies were included. We included studies from any developed country which is a member of the Organisation for Economic Collaboration and Development, as these have the most similar health delivery systems to the UK. We included studies published in English, and those in other languages which provided English abstracts.

### Data extraction and synthesis

Full copies of citations coded as potentially relevant were obtained, and those meeting the inclusion criteria were read in detail and data extracted. Three members of the research team carried out the screening and data extraction. The heterogeneity of the included work precluded summarising the studies via meta-analysis. Review findings were reported instead using narrative synthesis methods, and Harvest Plot techniques [18] to provide a visual summary of intervention effectiveness.

### Quality appraisal

Quality assessment of the effectiveness studies was based on an adaptation of the Cochrane criteria for judging risk of bias [19] due to the wide range of study designs. This method classifies studies in terms of sources of potential bias within studies: selection bias; performance bias; attrition bias; detection bias; and reporting bias. The completed assessment for each study is available as Additional file 1.

## Results of the review

**Fig. 1:**
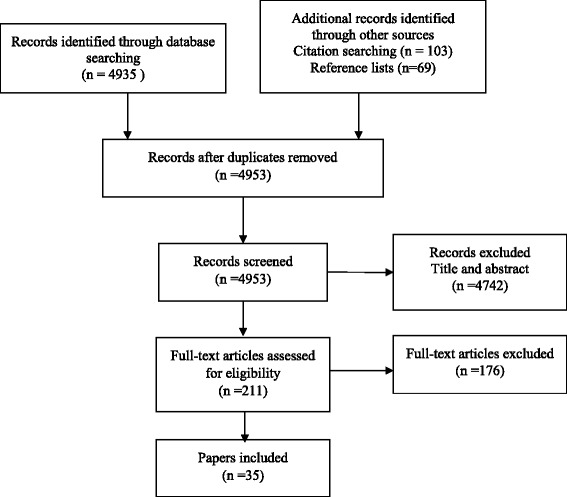
Process of selection of studies: first search

**Fig. 2:**
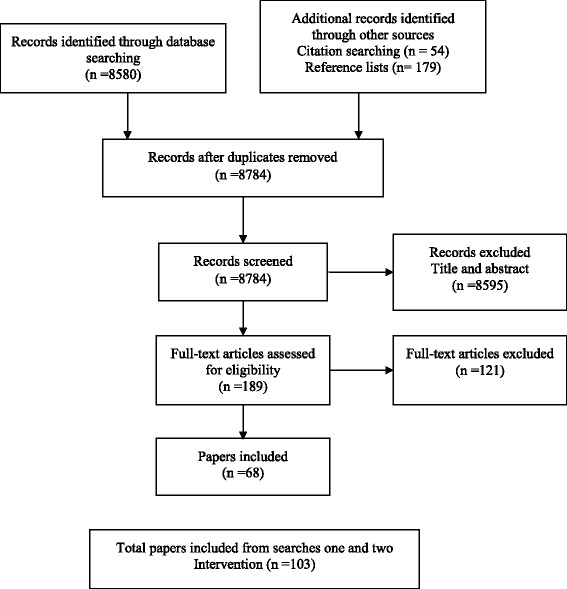
Process of selection of studies: second search

From a database of 13,253 citations, 103 papers were included in the review (see Figs. 1 and 2). Within this set of articles we found only one paper [20] which specifically referred to the participants as being recently retired. The literature provided predominantly age bands or average ages for their study populations. Some included numbers in employment/not in employment, and a few included the percentage who were retired. Tables listing papers by population characteristics are available as Additional file 1.

In the absence of literature referring to our target population, we used age as a proxy for the period of retirement transition. We developed a grading system of applicability for the papers, with A1 papers having populations described as recently retired or about to retire (*n* = 1), A2 papers with population mean or median of 55–69 years (*n* = 63), A3i papers had a population mean/median in the range of age 70–75 (*n* = 30), and A3ii papers had a population mean/median of age 49–54 (*n* = 9). In this article we focus on the A1/A2 studies [20–84].

The literature was of a reasonably high quality in terms of design, with a large proportion (*n* = 55) of the studies using a randomised or cluster randomised design. The most frequent areas of concern were: limited reporting regarding the process of randomisation; the recruitment of volunteer participants; studies having multiple intervention arms with no control condition; the wide use of self-reported data; and in some studies high rates of drop out.

The greatest proportion of work was reported by authors based in the USA (*n* = 32), followed by The Netherlands (*n* = 12) and then Australia/New Zealand (*n* = 9). Around a third of papers (*n* = 32) reported interventions with majority female participants. Few (*n* = 2) studies recruited only males. We identified only one study which described participants as being of predominantly low socio-economic status [61] and one paper in a minority ethnic population [69].

The studies measured a wide range of outcomes (a table of the outcomes reported is provided as Additional file 1). These included self-completed questionnaires (in person, via telephone or postal), outcomes that were measured by the research team (including weight, BMI, and fitness tests), together with data downloaded from pedometers or accelerometers. Two papers measured sedentary behaviour in addition to activity [55, 61].

**Fig. 3:**
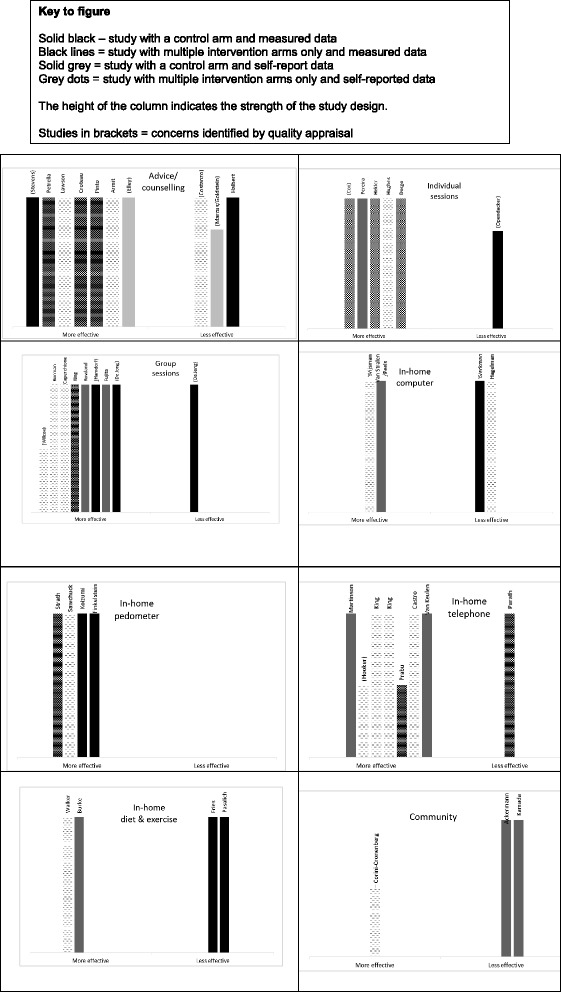
Harvest plot visual summaries

The studies reported a varied range of intervention approaches, which we classified into eight typologies. A summary of the characteristics of each study and main findings is available as Additional file 1. The Harvest plots (Fig. 3) provide a visual summary of the reported effectiveness of interventions within each typology. The categorisation as more/less effective was based on the proportion of outcome measures that were significantly different (*p* <0.05 or *p* <0.01), either from baseline to follow up (for those with multiple intervention arms only), or between intervention and control groups. To be considered “more effective”, the majority of outcomes (at least half) relating to PA needed to show a positive intervention effect.

In these charts each unique study is represented by a column. The height, shading and pattern of each column indicates the strength of the evidence in terms of type of study design, use or non-use of a comparator arm, and self-reported versus measured outcomes. Studies which can be considered to provide stronger evidence of effectiveness are those with solid black columns. Studies which can be considered to provide weaker evidence of effectiveness are those with grey, dotted columns.

As can be seen from the plots, the evidence regarding effectiveness for the different types of interventions is fairly positive across the set. Strength of evidence is best judged by considering not only volume of papers but also consistency of findings [18, 83]. Therefore, we outline both volume and consistency of study results below.

### Intervention typology

#### Counselling and advice

Eleven papers (10 studies) assessed the effectiveness of interventions comprising the giving of advice or counselling [21–31]. One of these interventions was delivered by peer mentors, one by trained physicians, one by a nurse, two by an exercise professional, two encouraged patients to prompt their physician, and the final four papers examined combined physician and exercise professional input. Seven of the advice/counselling interventions led to positive effects, although there is inconsistency in effectiveness, and only one study had a control group and measured outcomes. Only one study in this category had a follow up of more than 12 months.

#### Group sessions

Thirteen papers (nine studies) evaluated group-based programmes provided in community settings [32–44]. All but one of the studies seemed to result in positive effects, thus indicating strength in terms of consistency. Half of the studies had control-group designs and also two used objective measures indicating strength in terms of quality. The evidence for this typology comprised a total of seven studies and therefore suggests strength of evidence not only in terms of design but also in terms of number of studies of group interventions. One of the more effective studies had a follow up of more than 12 months, and all of the group interventions had a follow up period of at least 6 months, also suggesting the effectiveness of these interventions in terms of longer term change.

#### Individual exercise programmes

Seven papers (six studies) evaluated individual exercise programmes delivered either at home or in community settings [45–51]. All but one of the six studies reported that interventions were effective, although only one of these had a control arm. The single study (reported in two papers) suggesting less effectiveness was of a lower quality study design, although it had more than 12 months follow up. One of the studies suggesting greater effectiveness had more than 12 months follow up.

#### In-home telephone interventions

Nine papers (eight studies) evaluated in-home interventions which were predominantly delivered via the telephone [52–60]. Two of these studies used automated telephone contacts, rather than human coaches, and one paper reported evaluation of a smartphone application. All but one of the interventions appeared to be effective, with three of these having longer follow up periods. The study indicating less effectiveness was of the highest quality, although had only a brief follow up period.

#### In-home combined diet and exercise interventions

Six papers (four studies) outlined the results of interventions which targeted lifestyle more generally, and consisted predominantly of materials delivered to the home by post/mail [61–66]. The evidence for this typology was inconsistent, although the best quality studies suggested less effectiveness for PA outcomes.

#### Home-based interventions providing pedometer/accelerometer

While several of the studies reported in other groups included provision of a pedometer as part of the intervention element, four studies evaluated home-based interventions in which the provision of a pedometer/accelerometer was the core component [67–70]. The pedometer-based studies all provided evidence of effectiveness, with three of the studies including measured outcomes (steps) and two using control group designs. This suggests the strength of evidence for pedometer interventions in terms of consistency, although there are only four studies. All these studies had only short or immediate follow up, and therefore do not provide evidence of a longer term impact of these interventions. One found that pedometers did not produce superior results to advice-only interventions.

#### Computer-based interventions

Eleven papers (four studies) reported programmes using computers, with all incorporating web-based components [71–80]. One of these studies was the only paper we identified which referred to participants as recent retirees [20]. The evidence here was inconsistent, with equal numbers of papers suggesting both more and less effectiveness.

#### Community-wide initiatives

One randomised controlled trial aimed to enhance the content of health professional consultations [81]. Two studies evaluated community interventions (a publicity campaign, and free bus passes) [82, 83]. There was inconsistency in findings.

## Discussion

The majority of studies included in this review reported some intervention effect, although as many studies reported multiple outcomes including perceived change and readiness for change, this may be unsurprising. It is also important to note the tendency for improvement in participants over the period of the study, which may not be related to the intervention. This review echoes earlier studies in finding evidence of effectiveness for a range of interventions intending to increase physical activity in older adults [1, 6, 10]. While group-based, and home-based, with behavioural/activity and/or educational/cognitive components may all be of benefit, there may be differences in longer term effectiveness between components which requires further investigation.

Just under two thirds of the included intervention papers described a theoretical base for their study. These were mainly psychological theories such as stages of change, social cognitive theory, educational theories, or models specific to the motivation of active living. Few papers discussed how the chosen theory impacted on the results of their study. The impact of particular theoretical bases for interventions is an area that could be further explored in future research.

We found little evidence specifically referring to interventions during the retirement transition period despite the significance of this period of life change, and association between PA levels and retirement [1]. All but one of the studies we found set wide age ranges for inclusion, or included only populations of retired people. While there was no indication that these interventions would be unsuitable or not effective for people about to retire or recently retired, there is the potential that programmes might have differential effects in this specific population.

Difference in outcome between population sub-groups was rarely mentioned by authors, with little evidence regarding differential impacts on advantaged versus disadvantaged populations. The category of “older adults” used in many studies encompassed individuals at potentially very different stages of the life course, and consequently varying levels of health and physical ability. Further attention to effects on sub-groups of individuals within populations described as “older adults” seems required. Many of the studies were carried out with groups of predominantly female participants, and males may have differing preferences for, or response to particular types of intervention. Future research should explore the reasons for different changes in PA at retirement between different socio-economic groups, as these may hold the key to what interventions are most effective. If disposable income creates opportunities to be more active, and fatigue from chronic conditions reduces opportunities, then perhaps tackling lifestyle and chronic conditions much earlier than ‘retirement’ may be most important.

Our study builds on existing reviews by highlighting that evidence is needed regarding optimal interventions around this potentially significant point of life change. The wider evidence base suggests that some types of interventions are likely to be effective in this age group, and there is a striking evidence gap. Previous reviews have examined interventions for older adults more widely. Further development of effective and cost-effective approaches for this population using the evidence we already have, could potentially be a way to target individuals who might not seek out support to be more active in older age, but who might be receptive to an offer of support/activities at the time of retirement. Our review also builds on other reviews which have explored associations between disadvantage and activity [1, 17]. We highlight the need for greater exploration of differential response to PA interventions in population sub-groups.

The analysis of intervention effectiveness within and across typologies is adversely impacted by the diverse range of outcome measures currently in use. These include those which relate to levels of activity, levels of fitness, psychosocial elements, and correlates of physical activity. If the effectiveness of different interventions is to be compared, there needs to be greater agreement amongst researchers regarding key measures of change. Future studies should endeavour to include objective measures of activity, and not be reliant on self-report data, and include no-intervention rather than comparator intervention control arms, if the true impact of interventions is to be assessed.

### Limitations

Given the lack of studies which identified their population as being about to retire or recently retired, we developed an applicability rating which used age range as a proxy for retirement transition. We acknowledge that the mean or median ages reported by the studies may not reflect the true range of participant ages, and that age does not necessarily equate to retirement. The OECD reports [84] that the statutory retirement age for men and women in all but two member countries is 65 or 67. However, people may choose (or be forced by health or other circumstances) to retire earlier or later than this. Evidence suggests that people in low SES have more multiple morbidities (physical and mental health) so it may be that by excluding populations with specific conditions we missed studies involving low SES groups.

While the Harvest plot method of presenting the data provides a useful visual summary of effectiveness, we acknowledge that interpretation is complicated by several studies comparing different interventions, and often similar variants of intervention elements. The typology that we have adopted is also only one way of grouping the studies, and we recognise that alternative groupings are possible. This is particularly the case for those interventions with multiple elements.

## Conclusions

Studies are needed which are carried out specifically in adults in the period immediate before or shortly after retirement. This work is needed in order to evaluate whether existing interventions for older adults are most suitable and/or effective in adults who are about to retire or recently retired. Currently it is not known whether or not the retirement transition provides a key opportunity for interventions to effect change in physical activity levels throughout older life, and/or to reduce health inequalities.

## Additional file

Additional file 1: Summary of previous reviews, details of search strategy, and completed quality appraisals for included studies.(DOCX 74 kb)
