# Supplementary material for: Promoting and maintaining physical activity in the transition to retirement: a systematic review of interventions for adults around retirement age
Source: Int J Behav Nutr Phys Act. 2016 Feb 1;13:12. doi: 10.1186/s12966-016-0336-3 (PMC4735960; doi:10.1186/s12966-016-0336-3)
Supplement: Additional file 1: — Summary of previous reviews, details of search strategy, and completed quality appraisals for included studies.(DOCX 74 kb) [file 12966_2016_336_MOESM1_ESM.docx]

**Additional online material**

Summary tables

**Counselling/advice interventions**

| **Armit et al. 2005**  **Country**: Australia  **Study design:** RCT described as feasibility study  **Length of follow up:** 24 weeks | **Number of participants:** N= 28 (complete data for 22)  **Age**: 55-70 mean 64 (SD 4.8)  **Retirement**: 81% retired  **Gender:** 35% male  **Education**: NR  **Ethnicity**: NR  **Other inclusion/ exclusion criteria:** physically inactive | **Intervention content** Three intervention arms – advice from GP only (written physical activity prescription, booklet and brief advice), second group 15-20 minute counselling session, same booklet as group 1, diary for goal setting and monitoring and 3 telephone calls from exercise scientist over 12 weeks, and third group received counselling and support plus pedometer and advice centred on increasing steps  **Control condition if applicable:** None - three intervention arms  **Data collection methods**: Self report physical activity survey  **Outcome measures:** Total minutes of physical activity calculated using time spent in walking, moderate and vigorous activities | **Main findings**  Significant effect of intervention but no statistical difference between groups. At 12 weeks overall increase of 116 weighted minutes of physical activity per week p<0.001.  Decrease from end of intervention to 6 month FU with average decrease 60 weighted minutes. However the decrease predominantly in group 1. No precise detail of the 12 month data but assumed not statistically significantly different to baseline. |
| --- | --- | --- | --- |
| **Costanzo & Walker 2008**  **Country**: USA  **Study design:** RCT  **Length of follow up:** unclear | **Number of participants:** N=51  **Age**:50-65 years, mean 54/55.08 (SD 4.01/3.87)  **Retirement**: 1 individual not employed  **Gender:** All female  **Education**: 52% degree educated in intervention group, 80% control  **Ethnicity**: 100% white non-hispanic in intervention group (96% control)  **Other inclusion/ exclusion criteria:** screened using Stage of Exercise Behaviour Questionnaire eligible if at contemplation or preparation stage, English speaking, answered no to all questions on Physical Activity Readiness Questionnaire | **Intervention content** Behaviour counselling, using 5As (ask advise, assist, arrange, agree). 5 sessions over 12 weeks  **Control condition if applicable:** one session received  **Data collection methods**: questionnaires  **Outcome measures:** Barriers to self efficacy scale, social support and exercise survey (family support subscale and friend support subscale), Modified 7 day activity recall | **Main findings**  “The intervention did not directly affect physical activity but indirectly influenced it via self-efficacy”.  Significant difference between groups over time for self-efficacy for physical activity (p=0.002), a large effect size 0.19. This was due to decrease in control group with intervention group unchanged.  Family support for physical activity and friend support increased significantly in intervention group (0.030 ES 0.10 and p=0.019) and was unchanged in controls. Increase in family support was however associated with less physical activity. |
| **Croteau et al. 2014**  **Country**: USA  **Study design:** RCT (described as pilot)  **Length of follow up:** 12 months | **Number of participants:** N= 36 (21 completed)  **Age**: 51-81 mean 64 (SD 8.8)  **Retirement**: 16 employed  **Gender**: 21 female (of 28 completers)  **Education**: 16 completed some college education  **Ethnicity**: 27 White  **Other inclusion/ exclusion criteria:** Patients that professionals considered were inactive and would benefit | **Intervention content** Maine in Motion, 6 month programme based on social cognitive theory. Uses a manual, individual counselling sessions, pedometer, self-monitoring. Also “plus” programme where participants attended group meetings each month for first 6 months.  **Control condition if applicable:** Two intervention arms only no control  **Data collection methods**: Steps recorded based on pedometer readings  **Outcome measures:** Daily steps | **Main findings**  Baseline 4,236 mean daily steps, significant intervention effect (p=0.015 ES 0.611). Both groups had significant increase at 6 months with no significant difference between groups (p=0.151. Standard group increase of 18% and plus group increase of 53% at 6 months, 14% at 12 months for standard group and 40% at 12 months for plus. Fading of effect for standard group, effect significant only for plus group baseline to 12 months FU (ES 0.606) |
| **Elley et al. 2003**  **Country**: New Zealand  **Study design:** Cluster RCT  **Length of follow up:** 12 month | **Number of participants:** unclear  **Age**: 40-79 year old (mean 57.2 (10.8)).  **Retirement:** NR  **Gender:** 301 (67%) female  **Education**: 106 (24%) post high school qualification  **Ethnicity**: 354 (78%) European origin  **Other inclusion/ exclusion criteria:** sedentary | **Intervention content** General practitioners were prompted by the patient to give oral and written advice on physical activity during usual consultations. Exercise specialists continued support by telephone and post.  **Control condition if applicable:** Control patients received usual care.  **Data collection methods**: Self-reported questionnaire  **Outcome measures:** Change in physical activity, quality of life (SF-36cardiovascular risk blood pressure**).** | **Main findings**  Mean total energy expenditure increased by 9.4 kcal/kg/week (p=0.001) and leisure exercise by 2.7 kcal/kg/week (p=0.02) or 34 minutes/week more in the intervention group than in the control group (p=0.04).  The proportion of the intervention group undertaking 2.5 hours/week of leisure exercise increased by 9.72% (p=0.003) more than in the control group (number needed to treat=10.3).  SF-36 measures of self-rated “general health,” “role physical,” “vitality,” and “bodily pain” improved significantly more in the intervention group (p<0.05). |
| **Goldstein et al. 1999**  **Country:** USA  **Study design:** cluster RCT  **Length of follow up**: 6 weeks and 8 months | **Number of participants**: n=34 physicians, n=355 patients  **Age:** 50 and above, mean 65.6 (SD 9.1)  **Retirement:** 36% employed  **Gender:** 65% female  **Education**: mean 12 education years  **Ethnicity:** 97% White  **Other inclusion/ exclusion criteria:** too active, were not ambulatory, unable to provide information on the telephone | **Intervention content** Physically Active for Life Project (physical activityL).. One hour physician training session, physical activity counselling session provided to patients with follow up appointment 4 weeks later.  **Control condition if applicable**: no physician training, no follow up appointment, usual care.  **Data collection methods:** telephone interview with patients, questionnaire to physicians  **Outcome measures:** Stage of motivational readiness for physical activity, physical activity scale for the elderly**.** | **Main findings**  99% of patients received a written prescription at initial appointment, 77% at follow up.  At 6 weeks intervention group more likely to be in advanced stages of motivational readiness for physical activity than controls (Preparation or Action 89% versus 74% p<0.001 OR 3.56 CI 1.79-7.08). Effect however not maintained at 8 months.  At 6 weeks 27% of intervention group versus 21% controls met recommended guidelines for physical activity (no significant group difference). At 8 months this was 28% for intervention versus 23% controls (no significant group effect).  No significant change in physical activity scale between groups at either 6 weeks or 8 months. Both groups increased at 6 weeks and decreased by 8 months. |
| **Halbert et al. 2000**  **Country:** Australia  **Study design:** RCT  **Length of follow up**: 12 months | **Number of participants:** 299  **Age:** 60 or over, mean age 67  **Retirement:** NR  **Gender:** 48% female  **Education:** NR  **Ethnicity:** NR  **Other inclusion/ exclusion criteria:** sedentary adults | **Intervention content**  20 minute session with exercise specialist. Intervention group received individualised advice, pamphlet and a plan. Potential barriers discussed.  **Control condition if applicable:** received pamphlet on good nutrition during 20 minute session.  **Data collection methods:** questionnaire, activity log, accelerometer for subsample.  **Outcome measures:** exercise questionnaire, SF-36, blood pressure, weight, blood sample | **Main findings**  All self-report measures of physical activity increased pre-follow up for both groups (p<0.05). Significantly greater physical activity in intervention group versus controls on all measures except walking (P<0.05). Men reported higher rates and more vigorous exercise.  For the sample using accelerometer no difference between intervention and controls on this physical activity data.  Difference between groups in regard to intention to exercise (p<0.001). |
| **Lawton et al. 2009**  **Country**: New Zealand  **Study design:** RCT  **Length of follow up:** 2 years | **Number of participants:** n= 1089  **Age**: 58.9 (SD 7)  **Retirement:** NR  **Gender:** All female  **Education**: NR  **Ethnicity**: NR  **Other inclusion/ exclusion criteria:** not undertaking  30 minutes of moderate intensity physical activity on at least five days of the week | **Intervention content**  Brief physical activity intervention led by nurse with six month follow-up visit and monthly telephone support over nine months.  **Control condition if applicable:** no intervention  **Data collection methods**:  **Outcome measures:** Physical activity (achieving the recommended 150 minutes of at least moderate intensity physical activity) | **Main findings**  At baseline,10%of intervention participants and 11% of control participants were achieving 150 minutes of at least moderate intensity physical activity a week.  At 12 months rates increased to 43% and 30% and at 24 months to 39.3% and 32.8% (p<0.001), respectively. SF-36 physical functioning (p=0.03) and mental health (p<0.05) scores improved more in intervention compared with control participants, but role physical scores were significantly lower (p<0.01).  Mean physical activity levels, however, were higher (p=0.01) and a greater proportion reached the target of physical activity in the intervention group compared with the control group at 12 months (233 (43%) v 165 (30%), (p<0.001), with levels declining but still significantly different at two years (214 (39%) v 179 (33%), (p<0.001). |
| **Marcus et al. 1997**  **Country**: USA  **Study design:** controlled before and after, pilot study  **Length of follow up**: 6 weeks | **Number of participants:** N= 44  **Age**: Over 50, mean 67.1  **Retirement**: 55% employed  **Gender:** 72% female  **Education**: NR  **Ethnicity**: NR  **Other inclusion/ exclusion criteria:**  Active less than 3 times per week | **Intervention content** Physically Active for Life Project (physical activityL). 1.5 hour physician training session, physical activity counselling session provided to patients with follow up appointment 4 weeks later.  **Control condition if applicable**: usual care.  **Data collection methods:** telephone interview with patients, questionnaire to physicians  **Outcome measures:** Stage of motivational readiness for physical activity, physical activity scale for the elderly, acceptability and feasibility of intervention. | Patients in intervention group recollected more activity counselling than controls. 32% of controls recollected physician had spoken to them about physical activity, all in experimental group.  Physical activity SE scores increased mean 148 (SD 87) to mean 154 (SD 76) at 6 weeks while scores for controls remained little changed, however no significant difference in change between groups (t=0.9 p>0.005).  Adjusting for baseline variables suggested a 17.6 point difference intervention versus control in physical activity SE scores. |
| **Petrella et al. 2010**  **Country**: Canada  **Study design:** Cluster RCT  **Length of follow up:** 12 months | **Number of participants:** Healthy, community-dwelling n=360  **Age**: mean age 64.9 (7.1) years (range 55 to 85 years)  **Retirement:** 56% retired  **Gender:** 52% female  **Education**: 46% secondary, 17% college, 15% University  **Ethnicity**: NR  **Other inclusion/ exclusion criteria:** inactive lifestyle | **Intervention content**  Intervention physicians were trained to deliver a tailored exercise prescription and a trans-theoretical behaviour change counselling program.  **Control condition if applicable:** Control physicians were trained to deliver the exercise prescription alone.  **Data collection methods**: unclear  **Outcome measures:** Cardiorespiratory fitness, energy expenditure (7-day physical activity recall). | **Main findings**  Mean increase in pVO2 max was significant for both the intervention (3.02 [95% CI = 2.40 to 3.65] mL/kg/min) and control (2.21 [95% confidence interval 1.27 to 3.15] mL/kg/min) groups at 12 months (p<0 .001); however, there was no difference between groups. Women in the intervention group improved their fitness significantly more than women in the control group did (3.20 vs 1.23 mL/kg/min). The intervention group had a 4–mm Hg reduction in systolic blood pressure, while the control group’s mean reduction was 0.4 mm Hg (p<0.001). The mean (SD) energy expended significantly increased and was higher in the intervention group than in the control group (69.06 [169.87] kcal/d vs –6.96 [157.06] kcal/d, p<0.006). |
| **Pinto et al. 2005**  **Country**: USA  **Study design:** RCT  **Length of follow up:** 6 months | **Number of participants:** 100 primary care patients  **Age**: mean age 68.5 years  **Retirement:** NR  **Gender:** 63.2% female  **Education**: College education 57.9  **Ethnicity**: 14.7% minority  **Other inclusion/ exclusion criteria:** none | **Intervention content** Brief advice versus extended advice. Extended Advice group received three face-to-face physical activity counselling sessions with a health educator, physical activity prescriptions, 12 physical activity counselling phone calls, 12 physical activity tip sheets sent by mail.  **Control condition if applicable:** two interventions  **Data collection methods**: Self-reported and objective measures of physical activity  **Outcome measures:** Self-reported physical activity using the 7-Day Physical Activity Recall instrument and objective activity monitoring using Biotrainers | **Main findings**  At 3 months, using the 7-Day physical activityR data, the ExtAd group (n=49) reported an increase of 3.85 (SD=0.89) weekly kilocalorie expenditure in moderate-intensity physical activity versus an increase of 0.83 (SD=0.94) in the BriefAd group (n=44, F=5.20, p=0.03)  At 6 months, the effects were sustained: the increase from baseline levels was 4.19 (SD=0.81) in the ExtAd group versus 1.11 (SD=0.85) in the BriefAd group (F=6.62, p=0.05). |
| **Stevens et al. 1998**  **Country**: UK  **Study design:** RCT  **Length of follow up:** 8 months | **Number of participants:** n=714 inactive people  **Age**: mean age 59.1  **Retirement:** 55% economically active  **Gender:** 40% male  **Education**: 28% degree, 36% no qualifications  **Ethnicity**: 87% White  **Other inclusion/ exclusion criteria:** 55% sedentary | **Intervention content**  Consultation with an exercise development officer, and a personalised 10 week programme combining leisure centre and home based activities.  **Control condition if applicable:** Control subjects were sent information on local leisure centres.  **Data collection methods**: questionnaires  **Outcome measures:** physical activity, sedentary | **Main findings**  Net 10.6% (95% CI 4.5 to 16.9%) reduction in the proportion of people classified as sedentary in the intervention group compared with the control group, eight months after the intervention.  The intervention group also reported an increase in the mean number of episodes of physical activity per week, as compared with the control group (an additional 1.52 episodes (95% CI 1.14 to 1.95)).  The cost of moving a person out of the sedentary group was shown to be less than £650. The cost of moving someone to the now commonly recommended level was estimated at almost £2500 |

**Group session interventions**

| **Burman et al. 2011**  **Country**: USA  **Study design:** RCT  **Length of follow up:** 18 months | **Number of participants:** N=69  **Age**: 50 or older mean 63.42 (SD 8.62), 39% 50-64, 42% 65 or older  **Retirement**: NR  **Gender:** 82% female  **Education**: “primarily college educated”  **Ethnicity**: 91% White  **Other inclusion/ exclusion criteria:** currently inactive, free of medical factors prohibiting exercise or any other factors affecting study compliance or assessment | **Intervention content**  Two intervention arms **-** Active Adult Mentoring Program 16 weeks, a peer led advice and support group intervention or standard community group intervention. Both had access to exercise facility and given pedometer.  **Control condition if applicable:** Two intervention arms  **Data collection methods**: Questionnaires, treadmill assessment  **Outcome measures:** Leisure Time Exercise Questionnaire, a subsample used an accelerometer, cardiorespiratory fitness, Barriers self-efficacy and exercise self-efficacy, Exercise Motivation Scale. | **Main findings**  At end of the intervention both groups reported significantly more activity than at baseline (p<0.001 ES 1.38). No difference between the interventions. At 18 month FU the AAMP intervention group reported significantly more moderate to vigorous activity minutes per week than the usual intervention arm (p=0.04 ES 0.32).  Both groups improved equally on cardiorespiratory fitness (p=0.06 ES 0.63  Exercise related self-efficacy was not significantly different pre-post for either group.  Self-determination improved with the AAMP intervention, it was significantly more effective than the usual intervention (p=0.045 ES 0.46) |
| --- | --- | --- | --- |
| **Caperchione & Mummery 2006**  **Country**: Australia  **Study design:** RCT  **Length of follow up:** 12 months | **Number of participants:** N= 122  **Age**: Above 50, Mean age 58/59  **Retirement**: 38% retired, 50% employed  **Gender:** 82% and 66% female  **Education**: NR  **Ethnicity**: NR  **Other inclusion/ exclusion criteria:** history of inactivity (less than 15 minutes of physical activity), able to walk unassisted for 10 minutes | **Intervention content**  12 week lifestyle intervention. 5 enhanced groups and 5 standard groups met 1x week for 90 minutes over 12 week period. Instructor led group walk and group education sessions. Enhanced package included education sessions on group process.  **Control condition if applicable:** Two intervention arms only  **Data collection methods**: Questionnaires  **Outcome measures:** Community Activities Model Program for Seniors Questionnaire (CHAMPS) records energy expenditure of moderate activities, energy expenditure of total activities, frequency of moderate activities, frequency of total activities. Total expenditure of all activities (measured by calorific expenditure) chosen as the measure for this study.  Physical Activity Group Environment Questionnaire assessed perceptions of group cohesion. | **Main findings**  Significant increase in physical activity behaviour (as measured by calorific expenditure) over time for both groups (p<0.05). However no significant difference between standard and enhanced intervention.  Group cohesion measure decreased over time with no difference between the groups (was moderate to high across time points). |
| **De Jong et al. 2006**  **Country:** The Netherlands  **Study design:** cluster RCT  **Length of follow up:** 6 months | **Number of participants:** n=315  **Age:** 55-65 mean age 59.6 intervention, 58.8 controls.  **Retirement:** NR  **Gender:** 55% female  **Education:** 43%/33% elementary only  **Ethnicity:** NR **Other inclusion/ exclusion criteria:** none | **Intervention content**  Groningen active living model – 30 once a week one hour sessions emphasising moderate intensity recreational sport activities such as dance, self-defence, swimming. Sessions groups of 15-24 held in a gymnasium.  **Control condition if applicable**: waiting list for 6 months  **Data collection methods:** questionnaire, fitness testing session  **Outcome measures:** physical activity questionnaires used to estimate energy expenditure, vitality plus scale, quality of life, perceived fitness score, comparative fitness rating, physical activity readiness, blood pressure, body fat. | **Main findings**  Both groups significantly increased on virtually all measures however no difference between groups for most including energy expenditure (p=0.68), leisure time physical activity (p=0.8), and walking (p=0.78). Only significant difference between groups related to improved reach. ITT analysis of perceived fitness showed between group differences (p<0.01), also diastolic blood pressure (p<0.05). |
| **De Jong et al. 2007**  **Country: The Netherlands**  **Study design:** cluster RCT  **Length of follow up**: 12 months | **Number of participants:** n=181 (79 intervention)  **Age**: 55-65 no detail of this sample only baseline characteristics  **Retirement:** NR  **Gender:** baseline data only no detail for this sample  **Education:** as above  **Ethnicity:** as above **Other inclusion/ exclusion criteria:** as above | **Intervention content**  GALM – follow up data from De Jong et al. 2006  **Control condition if applicable**: as above  **Data collection methods:** as above  **Outcome measures:** as above | **Main findings**  Substantial amount of missing data (up to 49%)  Average attendance at sessions was 80% for first 6 months and 71% for second 6 months.  Energy expenditure for sport and total EE significantly increased over time - 12 months from baseline (p<0.01), however was not significant when compared by group. For EE leisure time activity, the intervention group while increasing to 6 months decreased 6 to 12 months. BMI & walking also significant effect by time but not group. Other indicators no significant differences by time or group. |
| **Fujita et al. 2003**  **Country**: Japan  **Study design: RCT**  **Length of follow up:** 6 months | **Number of participants:** N=65  **Age**: 60-81 years, mean age 67.1  **Retirement**: NR  **Gender:** 30 male, 35 female  **Education**: NR  **Ethnicity**: NR **Other inclusion/ exclusion criteria:** Those with neurological deficits, CHD, high blood pressure, arthritis, recent falls, chronic disease, other heart problems or conditions affecting participation | **Intervention content**  25 week exercise group intervention consisting of three two hour exercise classes each week. The classes consisted of endurance training, sit ups, and exercises using resistance bands.  **Control condition if applicable:** Received two x two hour classes per month consisting of a lecture on a non-physical activity topic and a recreational activity  **Data collection methods**: Physical activity diary  **Outcome measures:** Total daily energy expenditure, with activities classified as light, moderate or strenuous | **Main findings**  The total daily energy expenditure increased in the intervention group (from 40.8 to 43.5 kcal/kg per day, an increase of 6.4% p=0.03) and did not change in the control group (42.2 pre, 39.2 post p=0.11).  The light activity time in the intervention group decreased significantly (p=0.049) while both moderate and strenuous activity increased (although not significantly different p>0.05).  At follow up there was some fading of effect (decrease of 1.2 kcal/kg per day) however post intervention values remained higher than at baseline (p=0.05). Moderate activity rates remained significantly higher although strenuous activity had returned to pre-intervention level. |
| **Hamdorf et al. 1992**  **Country**: Australia  **Study design:** RCT  **Length of follow up:** 12 months (follow up data reported in paper below) | **Number of participants:** N=66  **Age**: 64.8 (60.6-69.2)  **Retirement:** NR  **Gender:** female  **Education**: NR  **Ethnicity**: NR  **Other inclusion/ exclusion criteria:** sedentary women | **Intervention content**  6 month (26 week) progressive walking programme. Two sessions per week supervised by fitness instructor.  **Control condition if applicable:** instructed to carry on with normal activities of daily living and not commence any new physical activity programmes.  **Data collection methods**: human activity profile, maximum current activity (MCA) and normative impairment index (NII)  **Outcome measures:** activity, aerobic fitness | **Main findings**  The maximum current activity score increased significantly (p=0.01) in the intervention group compared with the controls. |
| **Hamdorf et al. 1993**  **Country**: Australia  **Study design:** RCT  **Length of follow up:** 12 months | **Number of participants:** N=66  **Age**: 64.8 (60.6-69.2)  **Retirement:** NR  **Gender:** All female  **Education**: NR  **Ethnicity**: NR **Other inclusion/ exclusion criteria:**  Sedentary women | **Intervention content** 6 month (26 week) progressive walking  programme. Two sessions per week.  **Control condition if applicable:** Instructed to carry on with normal activities of daily living and not commence any new physical activity programmes.  **Data collection methods**: Human activity profile, maximum current activity (MCA) and normative impairment index (NII)  **Outcome measures:** activity, aerobic fitness | **Main findings**  During the follow up period 77.8% of the training group continued with exercise and maintained lower (p<0.005) exercise heart rates compared to the control group.  The training group had significantly higher maximum current activity scores than the controls at the end of the training period which were maintained during the six month follow up (p>0.005) |
| **King et al. 2000**  **Country**: USA  **Study design:** RCT  **Length of follow up:** 1 year | **Number of participants:** 103 older adults  **Age**: 65+ mean 69 (SD 4)  **Retirement:** NR  **Gender:** 67 women; 36 men  **Education**: NR  **Ethnicity**: 95% White  **Other inclusion/ exclusion criteria:** regularly active no more than twice a week during the preceding 6 months | **Intervention content** Randomized to 12 months of community-based, moderate-intensity endurance and strengthening exercises (Fit & Firm) or stretching and flexibility exercises (Stretch & Flex). A combination of class and home-based exercise formats.  Exercise classes lasted one hour, and the duration of the home-based sessions was 40 minutes.  **Control condition if applicable:** Two intervention arms  **Data collection methods**: Exercise logs  **Outcome measures:** Physical performance, perceived functioning and well-being (self-rated) | **Main findings**  For both conditions, exercise adherence was significantly higher for the home-based portion of the exercise prescription (Fit & Firm: 92 ±50%; Stretch & Flex: 92 ±43%) relative to the class-based portion of the exercise prescription (Fit & Firm: 65 ±27%; Stretch & Flex: 68 ±29%). For home versus class comparison: paired-comparison t test=5.2, p<0.001, two-tailed test.  Overall between-group differences on the two measures constituting the self-rated physical performance domain—including the three subscales contained in the Colorado Walking Impairment Scale and the four subscales contained in the Self-Efficacy for Physical Performance Scale was significant (F[7,99] = 2.46, p<0.01, one-tailed test). |
| **Rowland et al. 1994**  **Country**: UK  **Study design:** RCT  **Length of follow up:** mean 10 months (range 3 to 20 months) | **Number of participants:** 739 women  **Age**: mean age of 66  **Retirement:** All retired  **Gender:** Female  **Education**: NR  **Ethnicity**: NR **Other inclusion/ exclusion criteria:** none | **Intervention content**  Programme taught for two hours a week by a trained Health Education Authority teacher over 10 weeks. Combined both exercise theory and practice. **Control condition if applicable:** No intervention  **Data collection methods**: Nottingham Health Profile questionnaire  **Outcome measures:** Exercise knowledge, attitudes, and behaviour | **Main findings**  The intervention group had significantly better self-perceived health, indicated by lower Nottingham Health Profile scores, more exercise knowledge, and positive exercise attitudes, and more exercise than the controls.  The mean number of hours spent on domestic activity and sport/recreational exercise per week were both greater in the intervention group (13.3 vs. 12.5 hours F=2.1 p=0.11 and 5.0 vs. 3.9 hours F=9.5, p<0.001). |
| **Wilcox et al. 2006**  **Country**: USA  **Study design:** Before and after  **Length of follow up:** post intervention (6 months) | **Number of participants:** n=838  **Age**: 68.4 ±9.4 years  **Retirement:** NR  **Gender:** 80.6%  women  **Education**: NR  **Ethnicity**: 64.1% White.  **Other inclusion/ exclusion criteria:** none | **Intervention content**  Active Choices (AC), a 6-month, telephone-based program, and Active Living Every Day (ALED), a 20-week, group-based program.  **Control condition if applicable:** Two intervention arms  **Data collection methods**: Pre and post-test surveys  **Outcome measures:** Physical activity (moderate to vigorous and total physical activity) | **Main findings**  Participants reported a significant increase from pre-test to post-test in moderate- and vigorous-intensity physical activity (t10=11.67; p<0.001) and total physical activity (t10=13.53; p<0.001).  Participants reported improvements in satisfaction with body appearance (t10=11.22; p<0.001), body function (t10=10.39; p<0.001), depressive symptoms (t10=–3.14; p<0.05), and perceived stress (t10=–2.90; p<0.05). BMI decreased (t10=–5.19; p<0.001).  Effects were statistically significant for both types of programme. |
| **Wilcox et al. 2008**  **Country:** USA  **Study design:** Follow up survey as part of above study  **Length of follow up**: 4 years | **Number of participants:** Active choice n=2503  Active living every day n=3388  **Age**: 50 or over, average 65.8 years AC and 70.6 years  **Retirement:** NR  **Gender:** 80% female AC and 83% ALED  **Education**: less than high school 8/9%.  **Ethnicity**: AC 41% non Hispanic White ALED 64% non-Hispanic White **Other inclusion/ exclusion criteria:** none | **Intervention content**  As above  **Control condition**  Two intervention arms  **Data collection methods**: Survey  **Outcome measures:** CHAMPS physical activity questionnaire, Depression Scale, Perceived Stress Scale, body satisfaction | **Main findings**  Same pattern in years 3 and 4 as that for year 1 reported in above paper. ALED all outcomes significantly changed except for depressive symptoms (approached significance). AC all outcomes significantly changed except for perceived stress.  For both programmes the proportion of participants reaching exercise recommendations increased significantly.  AC year 1 p=0.001, year 2 p<0.001, p<0.001.  ALED year 1 p<0.001, p<0.001, p<0.001. |
| **Wilcox et al. 2009**  **Country**: USA  **Study design:** Before and after  **Length of follow up:** immediate | **Number of participants:** N= 1955 provided data, all in year one and first 100 in year 3 asked to provide baseline/Fu data. The paper describes target recruitment of 8,100, not clear the total number receiving the intervention.  **Age**:50 or older, Active Choices 43.7% aged 50-64 and 35% 65-74, Active living every day 28.8% 50-64 and 37.6% 65-74. Mean 68.4 (SD 9.4)  **Retirement**: NR  54.3%/46.7% under 30,000 annual income  **Gender:** 79.1%/82.5% female  **Education**: Active choices 63% some college or higher, Active living every day 61% some college or higher  **Ethnicity**: Active choices White 41.8%, Black or African American 36.4%. Active living every day 62.8% White, 30.4% African American.  **Other inclusion/ exclusion criteria:** underactive adults (physical activity less than two days a week) | **Intervention content**  Active for life programme – two interventions within this programme evaluated in this paper. Active choices – over 6 months one in-person orientation session followed, by 8 telephone counselling sessions. Active living every day – 20 week intervention delivered by weekly sessions in small groups.  **Control condition if applicable:** Two intervention arms  **Data collection methods**: Questionnaires  **Outcome measures:** Physical activity self-efficacy scale, a social support scale, Center for Epidemiological Studies Depression Scale, Perceived Stress Scale, CHAMPS questionnaire (Community Healthy Activities Model Program for Seniors), a 3 item measure from the Behavioural Risk Factors Surveillance system**.** | **Main findings**  Findings reported predominantly by predictors of greater or lesser change rather than effectiveness. Effect size of 0.8 for 55-64 year olds for Active choices and ES 0.87 for active living. Little difference ethnicity (White 0.56 and 0.67 versus Black African American 0.62 and 0.72). Active choices greater effect in those reported as receiving less than high school education than those reporting receiving education to high school or more than high school (0.79, 0.58. 0.64). |
| **Wilcox et al. 2009**  Country: USA  **Study design:** follow up survey as part of above study  **Length of follow up:** 6 months after completing the programme | **Number of participants:** unclear n=169?  **Age**: mean age 72 by time of this data collection phase  **Retirement:** NR  **Gender:**72% women  **Education**: 64% college or greater  **Ethnicity**: 26% non-Hispanic White **Other inclusion/ exclusion criteria:** as above | **Intervention content**  As above  **Control condition if applicable:** Two intervention arms  **Data collection methods**: Questionnaire  **Outcome measures:** Behavioural Risk Factors Surveillance System, satisfaction with body functioning | **Main findings**  Active Choices – time effects significant for physical activity (p<0.0001/p<0.05). Improvements post-test were maintained at 6 month FU.  Active Living Every Day – year 3 time effects significant (p<0.0001/p<0.001). A significant decline from post-test to FU for physical activity, although values remained higher than at baseline. |

**Individual exercise programme interventions**

| **Cox et al. 2008**  **Country**: Australia  **Study design:** RCT  **Length of follow up:** 12 months | **Number of participants:** N= 116  **Age**:50-70 years, mean age 55 years  **Retirement**: 52%-80% employed  **Gender:** All female  **Education**: 13-15 years education  **Ethnicity**: Described as predominantly well-educated Caucasian women **Other inclusion/exclusion criteria:** non-smokers, less than 30 mins of activity per week, BMI <34kg/m2. | **Intervention content**  SWEAT behavioural intervention. consisted of workshop centre-based sessions with worksheets to complete at home, supervised swimming or walking programme, followed by unsupervised programme  **Control condition if applicable:**  Four study arms – swimming and usual care, swimming and SWEAT, walking and usual care, walking and SWEAT  **Data collection methods**: Baseline measures and reassessment at 6 and 12 months  **Outcome measures:** Stage of Change Instrument, exercise history questionnaire, 1.6km walk, 12 minute swim, record of illness and injury. | **Main results relevant to research question (author analysis)**:  After 6 months 83% of swimmers and 86% of walkers had moved to action stage of stages of change. After 12 months 75% of swimmers and 72% of walkers had moved to maintenance stage. Similar in control and intervention groups. Benefits in terms of fitness (walk time or swim distance) found for both groups at 6 months and were maintained at 12 months compared to baseline (p<0.05). |
| --- | --- | --- | --- |
| **Dorgo et al. 2009**  **Country**: USA  **Study design:** RCT  **Length of follow up:** 14 weeks | **Number of participants:** 60 older adults  **Age**: Mean age 68.7 (60-82 years)  **Retirement:** NR  **Gender:** 31 men, 29 women.  **Education**: NR  **Ethnicity**: NR **Other inclusion/ exclusion criteria:** None | **Intervention content**:  30 older adults (mean age 68.4) trained as peer mentors. Identical 14 weeks fitness programme delivered by peer mentors (PM) or student mentors (SM).  **Control condition if applicable:** Student mentor delivered fitness programme  **Data collection methods**: Unclear  **Outcome measures:** Physical improvement, programme perception, retention and participation. | **Main findings**  Both groups improved their fitness significantly with no post-test differences between the groups in most fitness measures or programme perception rates. Significant improvements (p<0.007, range of ES 0.2-1.6) in all measures of fitness for the PM group. The same was true for the SM group (p<0.31, range of ES 0.2-1.4) with the exception of upper body flexibility (p=0.76). |
| **Hekler et al. 2012**  **Country**: USA  **Study design:** Cross over RCT  **Length of follow up:** Immediate | **Number of participants:** N= 16  **Age**:50 or older, mean 64 years (SD 7.5)  **Retirement**: 56% employed (no details of non-employed)  **Gender:**81% female  **Education**: 56% degree or higher  **Ethnicity**: NR **Other inclusion/ exclusion criteria:** inactive (less than 60 minutes a week of activity) | **Intervention content**  Two intervention arms - utilitarian walking (purpose of getting somewhere or accomplishing errands) and leisure walking (purpose of fitness health or recreation). Two weeks of each intervention, free choice for a further 2 weeks.  **Control condition if applicable:** Two intervention arms  **Data collection methods**: Pedometer, self-report of type during free choice.  **Outcome measures:** Number of steps per day | **Main findings**  Significant mean improvement in amount of walking for all 2 week phases (p<0.05). Significantly more mean steps per day during leisure intervention than utilitarian phase (p<0.05). Neighbourhood characteristics associated with walking type. |
| **Hughes et al. 2009**  **Country**: USA  **Study design:** RCT  **Length of follow up:** 10 months | **Number of participants:** 544 older adults  **Age**: 50 years or older, mean  age 66 years  **Retirement:** NR  **Gender:** 76.6% Female  **Education**: 86.6% college education  **Ethnicity**: 85.5% White **Other inclusion/ exclusion criteria:** 72% were overweight or obese | **Intervention content**  Multiple-component physical activity program that provided flexibility activities, low-impact aerobic exercise, and resistance training  **Control condition if applicable:** Control group participants could enrol in any programs except for the multicomponent program,  **Data collection methods**: Questionnaire and clinical measures  **Outcome measures:** self-efficacy for exercise adherence, exercise participation | **Main findings**  Statistically significant benefits for intervention group at 5 and 10 months with regard to self-efficacy for exercise adherence over time (p<0.001), adherence in the face of barriers (p=0.01), increased upper- and lower-body strength (p=0.02, p==.01), and exercise participation (p=0.01). |
| **Opendacker et al. 2011**  **Country:** Belgium  **Study design:** Controlled before and after  **Length of follow up:** 2 years | **Number of participants: n=186**  **Age:** 60-83 mean 66  **Retirement:** All retired  **Gender:** 50% male  **Education:** : Average 12-13 years education  **Ethnicity:** NR **Other inclusion/ exclusion criteria:** not being physically active, no medical problems contra-indicating participation | **Intervention content**  As above  **Control condition if applicable:**  Two intervention arms  **Data collection methods:** As above  **Outcome measures:** As above | **Main findings**  Improvements in cardiorespiratory fitness for both intervention groups however pre-test to follow up all groups including control had improved with no significant difference between them. |
| **Opendacker et al. 2008**  **Country:** Belgium  **Study design:** Controlled before and after  **Length of follow up:** 2 years | **Number of participants: n=186**  **Age:** 60-83 mean 66  **Retirement:** All retired  **Gender:** 50% male  **Education:** : Average 12-13 years education  **Ethnicity:** NR **Other inclusion/ exclusion criteria:** not being physically active, no medical problems contra-indicating participation | **Intervention content**  As above  **Control condition if applicable:** no intervention  **Data collection methods:** As above  **Outcome measures:** As above | **Main findings**  Both groups had significantly increased total physical activity compared to controls immediate post-test. Lifestyle intervention group significantly larger increases in active transportation and total steps compared to controls and the structured intervention arms at follow up.  Participtants who dropped out were significantly older and less educated than those who completed follow up. |
| **Pereira et al. 1998**  **Country**: USA  **Study design:** RCT  **Length of follow up:** 10 years | **Number of participants:** 196  **Age**: 50 to 65 years at baseline mean 57.8 (SD 4.29)  **Retirement:** NR  **Gender:** All female  **Education**: NR  **Ethnicity**: White **Other inclusion/ exclusion criteria:** post-menopausal, not taking hormone replacement therapy | **Intervention content**  Walking intervention (no details in this paper).  **Control condition if applicable:** no details in this paper  **Data collection methods**: Telephone interview  **Outcome measures:** Self-reported walking for exercise and other purposes, Paffenbarger sports and exercise index | **Main findings**  Median values for both usual walking for exercise (1000 vs. 302 kcal/wk) and total walking (1344 vs. 924 kcal/wk) were significantly higher for walkers compared to controls. (p=0.01 for both).  Compliers (walking at least 7 miles/wk during the intervention period) reported significantly more walking that all other groups at 10 years (p<0.05). |

**In-home telephone interventions**

| **Castro et al. 2001**  **Country**: USA  **Study design:** RCT  **Length of follow up:** 2 years | **Number of participants:**  179 (140 second year)  **Age**: 50-65, mean 56.3 (4.3)  **Retirement:** NR (74% employed)  **Gender:** 57% male  **Education**: 9-22 years, mean 15.6 (2.7) years  **Ethnicity**: 93% White **Other inclusion/ exclusion criteria:** none | **Intervention content**  Randomised to one year of telephone counselling to adopt higher (more vigorous) or lower intensity physical activity. After 1 year, randomised to second year of contact via telephone/mail or mostly mail only.  **Control condition if applicable:** Two intervention arms  **Data collection methods**: Unclear  **Outcome measures:** Self-reported exercise adherence rate. | **Main findings**  During year 1 participants in the higher condition had average exercise adherence of 88% (2.64 sessions per week), whereas the low condition averaged 81% (4.05 sessions per week).  During year 2, the higher condition had average exercise adherence of 73% (2.19 sessions per week), whereas the low condition averaged 57% (2.85 sessions per week).  When controlling for year 1 adherence rates, participants in the lower intensity programme did not differ from the higher intensity programme in their year 2 adherence rate F(1,63)=0.07, p>0.7 |
| --- | --- | --- | --- |
| **Hooker et al. 2005**  **Country:** USA  **Study design:** before and after  **Length of follow up:** 1 year | **Number of participants: n=447**  **Age:** 48-90, mean 67.9 (SD 8.6)  **Retirement**: 49% retired no work, 17.7% retired, working, 20.9% employed  **Gender:** 78% female  **Education:** 13% below High School, 17.9% income less than $10,000  **Ethnicity:** 58% Caucasian, 25% Hispanic **Other inclusion/ exclusion criteria:** NR | **Intervention content**  Participants met with staff member to develop a physical activity plan with individual goals. Received regular telephone calls over 1 year from a “buddy”. Variation in other elements between sites, some newsletters, some group activities, some face to face visits in addition to telephone support.  **Control condition if applicable:** None  **Data collection methods:** Questionnaires  **Outcome measures:** Physical Activity Readiness Questionnaire, calories, CHAMPS questionnaire | **Main findings**  Significant improvement in total calorie expenditure baseline to midpoint and endpoint (p<0.0001 median change 707 calories per week), a typical increase of 3.2% of calories expended per month. Those with lower levels at baseline and older than 65 tended to increase more.  Light and moderate activity levels showed similar improvement (p<0.0001 pre-post).  Improvement in stage of readiness for all stages. |
| **King et al. 2007**  **Country**: USA  **Study design:** RCT  **Length of follow up:** 12 months | **Number of participants:**  N = 218  **Age**: 55 years and older  **Retirement:** NR  **Gender:** NR  **Education**: NR  **Ethnicity**: NR **Other inclusion/ exclusion criteria:** not initially engaged in more than 60  minutes per week of moderate-intensity or more vigorous physical activity over the previous 6 months | **Intervention content**:  1) a home-based moderate-intensity physical activity program delivered primarily via a trained telephone counsellor (Human Advice arm);  2) a home-based moderate-intensity physical activity program delivered primarily via an automated, computer controlled interactive telephone system (Automated Advice arm)  **Control condition if applicable:** Weekly health education classes  **Data collection methods**: 7-day physical activity recall interview; verified on a random subsample via accelerometer data  **Outcome measures:** Minutes of moderate to vigorous physical activity. | **Main findings**  At 6 months, both the Human Advice and Automated Advice arms, while not significantly different from one another (p=0.73), had significantly greater mean energy expenditure in MOD activity than the Control arm (F(4, 217) 4.73, (p=0.01).  Similarly, both the Human Advice and Automated Advice arms, while not significantly different from one another ( p=0.65), had significantly greater mean minutes/week spent in MOD activity than the Control arm, F(4, 217) 4.73, p=0.01.  These differences were generally maintained at 12 months. |
| **King et al. 2013**  **Country:** USA  **Study design:** RCT  **Length of follow up:** Immediate | **Number of participants**: n=68  **Age:** 45-81, mean 59 (SD 9.2)  **Retirement:** 48.5% working full time  **Gender:** 73.5% female  **Education:** 66% college degree, 51.4% income $70,00 or more  **Ethnicity:** 69% non-Hispanic White **Other inclusion/ exclusion criteria:** insufficiently inactive, using a mobile phone but not a smart phone | **Intervention content**  **3** Smartphone Apps – one based on an analytical motivational frame which included goal setting, behavioural feedback, problem solving, tips and advice. Second based on social motivational frame including social normative feedback, social support, interacting with other and group based competition and collaboration. Third based on affective motivational frame including positive reinforcement, use of an avatar to provide feedback, and game like rewards for reaching milestones. Eight weeks intervention  **Control condition if applicable:** Three intervention arms  **Data collection methods:** Questionnaire  **Outcome measures:** CHAMPS questionnaire, measure of older adults sedentary behaviour, satisfaction survey | **Main findings**  Significant increases in minutes of brisk walking for all groups pre-post p<0.0001 with no difference between groups (p>0.73). Increase averaged 100.8 weekly minutes (SD 167).  Similarly increase in moderate to vigorous physical activity for all groups (p<0.0001) with no difference between groups (p>0.99). Decreases in reported sitting time, television watching. |
| **Martinson et al. 2008**  **Country**: USA  **Study design: RCT**  **Length of follow up:** 6 month outcomes | **Number of participants:** N= 1049  **Age**: 50-70 mean 57.1 (SE 0.2)  **Retirement**: 77% employed  **Gender:** 72% female  **Education**: 67% 4 year degree or more  **Ethnicity**: 92% White intervention group  **Other inclusion/ exclusion criteria:** as above | **Intervention content**  Keep Active Minnesota Two year interactive telephone and mail based support programme. 7 session course delivered approximately bi-weekly over the telephone by exercise coaches, then monthly calls for one year and then bi-monthly for the second year.  **Control condition if applicable:** Usual care – received information about the program and 4 newsletters over the 2 years.  **Data collection methods**: Self-report data collected via telephone using CHAMPS questionnaire  **Outcome measures:** Kilo calories expended per week in physical activity specifically during moderate to vigorous physical activity. | **Main findings**  At 6 months intervention versus control higher p=0.03 for all physical activity  For MV physical activity kcal expenditure intervention group higher p=0.03  For physical activity maintenance intervention group higher p=0.03. |
| **Martinson et al. 2010**  **Country**: USA  **Study design: RCT**  **Length of follow up:** 2 years | **Number of participants:** N= 1049  **Age**: 50-70 mean 57.1 (SE 0.2)  **Retirement**: 77% employed  **Gender:** 72% female  **Education**: 67% 4 year degree or more  **Ethnicity**: 92% White intervention group  **Other inclusion/ exclusion criteria:** included those reporting at least 30 minutes of moderate or vigorous physical activity at least 2 days per week and that they had increased their physical activity over previous 12 months. | **Intervention content**  As above  **Control condition if applicable:** As above  **Data collection methods**: As above  **Outcome measures:** As above | **Main findings**  Significantly more activity reported by intervention group than controls at 6 and 24 months (p<0.03 and p<0.01) ES d=10 -0.17. The intervention led to an increase in energy expenditure of around 200 kcalones per week extra equivalent to one hour per week of moderate intensity walking.  More intervention than control participants maintained MVphysical activity at 6, 12 and 24 months (p<0.003, p<0.004, p<0.001). At 24 months, while the usual care group dropped back to their 6 month level the intervention group continued to increase (p<0.05). |
| **Prabu et al. 2012**  **Country**: USA  **Study design:** before and after  **Length of follow up:** Immediate | **Number of participants:** n=85  **Age:** average age 57  **Retirement: NR**  **Gender:** all female  **Education:** 75% had a college degree, more than half annual income greater than $75000  **Ethnicity: 93%** White **Other inclusion/ exclusion criteria:** post-menopausal, BMI of 25-40 kg/m, access to mobile phone, willing to walk 30 minutes per day. | **Intervention content**  12 week walking programme. Daily steps goal and wore pedometer. Goals increased each week. Feedback regarding number of steps provided each week.  No coach condition - Each day for 12 weeks 2 contacts with an interactive voice response system  Coach condition – a trained coach available, explained the intervention and offered step goals. Trained participant to use pedometer and system and offered support during intervention.  **Control condition if applicable:** Two intervention arms  **Data collection methods:** Baseline data collected at visit to a gym, pedometer, questionnaires  **Outcome measures:** Exercise goal questionnaire, exercise planning questionnaire, exercise thoughts and barriers, social support, self-efficacy, waist and hip measurements, one mile walk. | **Main findings**  Significant decrease in one mile walk time pre-post p=0.001 d=0.41.  Change in goal setting, exercise planning, managing negative thoughts approximately effect size 0.8. Effect sizes for BMI, weight and waist measurement were small (0.07 to 0.14).  No between-group differences. |
| **Purath et al. 2013**  **Country**: USA  **Study design:** RCT  **Length of follow up:** Immediate | **Number of participants:** N= 72  **Age**: 60-80 mean age 66.2 (SD 5.2)  **Retirement**: NR  **Gender:** 53 women and 19 men  **Education**: Mean 2 years post high school  **Ethnicity**: NR **Other inclusion/ exclusion criteria:** Excluded people who were already active (more than 150 minutes of moderate activity per week) | **Intervention content**  Delivered by a nurse practitioner. Fitness testing followed by discussion of results and completion of How ready are you to change your physical activity survey, physical activity goals set for 2 weeks on a written prescription, asked to complete activity diary. Follow up phone call 2 weeks, with 10 further calls over 6 month intervention period.  **Control condition if applicable:** Received diet intervention consisting of initial questionnaire, goal setting, phone calls relating to healthy eating, fitness test results not discussed.  **Data collection methods**: Questionnaires, fitness testing  **Outcome measures:** CHAMPS questionnaire (calorific expenditure/frequency of moderate activity), Compendium of Physical Activity Tracking Guide, a subgroup of participants wore accelerometers, Senior Fitness tests of muscle strength, endurance and balance, BMI | **Main findings**  Intervention group and control group both increased weekly estimated calorific expenditure in all physical activity at 3 months and 6 months. The intervention group had a larger increase but this was not significant.  No significant change in weekly frequency of activity or measures of physical fitness in either group. Also no change in self-efficacy or barriers.  When controlled for baseline co-variates (age, gender, income, BMI, support) intervention participants significantly increased frequency of all physical activity compared to control (p<0.05) indicating the intervention was effective for selected participants. |
| **Van Keulen et al. 2011**  **Country**: Netherlands  **Study design:** RCT  **Length of follow up:** 73 weeks (baseline, 25, 47, and 73 weeks). | **Number of participants:** n=1,629  **Age**: 57.15 years (SD=7.13)  **Retirement:** NR  **Gender:** 55% male  **Education**: 54% low, 23% intermediate education level  **Ethnicity**: NR **Other inclusion/ exclusion criteria:** physically active with moderate intensity for mean 4.65 h a week (SD=3.83). | **Intervention content**  Participants randomly received either four tailored print letters, four motivational telephone calls, two of each type of intervention, or no information.  **Control condition if applicable:** No intervention  **Data collection methods**: telephone interviews/postal questionnaire  **Outcome measures:** Absolute change in self-reported physical activity and fruit and vegetable consumption | **Main findings**  All three intervention groups (tailored letters, motivational calls, and the combined version) were equally and significantly more effective than the control group in increasing physical activity (hours/day) from baseline to the intermediate measurement (week 25), follow-up 1 (week 47) and 2 (week 73). Effect sizes (Cohen’s d) ranged from 0.15 to 0.18. |

**In home combined diet and exercise interventions**

| **Burke et al. 2013**  **Country**: Australia  **Study design:** RCT  **Length of follow up:** Immediate | **Number of participants:** N= 375  **Age**: 60-70 mean 65 (SD 2.95 and 3.19)  **Retirement**: 43.8%/40.2% working  **Gender**: Male 52%/50%  **Education**: University 21%/17.6%  **Ethnicity**: NR **Other inclusion/ exclusion criteria:** classified as low or medium SES, insufficiently active (less than 30 mins of moderate-intensity physical activity on at least 5 days per week) | **Intervention content**  Physical activity and nutrition for seniors (physical activity NS) 6 month programme. Flexible home based, set own goals and semi-tailor own intervention. Comprised a booklet with recommendations and encouraging goal setting, also an exercise chart, calendar, bi-monthly newsletters, a resistance band and a pedometer. Telephone calls and email contact according to participant request. Included both activity and dietary elements.  **Control condition if applicable:** Completed questionnaires only and received small financial reward.  **Data collection methods**: Self-completed questionnaires  **Outcome measures:** International Physical Activity Questionnaire Short Form | **Main findings**  Significant improvements baseline to post-programme for intervention group in regard to strength exercise (P<0.001), walking (p=0.012), moderate activity (p=0.008), vigorous activity (p=0.044), sitting time mean per week (p<0.001).  These improvements were significant compared to controls strength (p<0.001) walking (p=0.0029) vigorous activity (p=0.0015) mean sitting time (p=0.0001). |
| --- | --- | --- | --- |
| **Fries et al. 1992**  **Country:** USA  **Study design:** cluster RCT  **Length of follow up:** 1 year | **Number of participants:** n=4712  **Age:** mean 68  **Retirement:** All retired  **Gender:** 52% female  **Education:** NR  **Ethnicity:** NR **Other inclusion/ exclusion criteria:** Members of bank retiree clubs | **Intervention content**  Personal health risk report based on questionnaire data every 6 months, individualised recommendation newsletters, newsletters, self-management and health promotion books, other materials all delivered by post.  **Control condition if applicable:** 2 other conditions – questionnaire only and no intervention  **Data collection methods:** Health habits questionnaire, health benefit company data on claims made.  **Outcome measures:** Health claims data, health risky behaviours including exercise minutes/week and health programme attendance, weight computed as health risk score. | **Main findings**  Computed health risk scores decreased by 4.3% in intervention group and increased by 7.2% in questionnaire only group.  Exercise minutes per week and exercise programme attendance non-significant difference baseline to 12 months or difference intervention versus controls.  Other health risk factors such as pounds over ideal weight, blood pressure and total health risk score were significantly different baseline-post and between groups. |
| **Fries et al. 1993**  **Country:** USA  **Study design:** cluster RCT  **Length of follow up:** 2 years | **Number of participants:** n=4712  **Age:** mean 68  **Retirement:** All retired  **Gender:** 52% female  **Education:** NR  **Ethnicity:** NR **Other inclusion/ exclusion criteria:** Members of bank retiree clubs | **Intervention content**  As above  **Control condition if applicable**: As above  **Data collection methods:** As above  **Outcome measures:** As above | **Main findings**  Computed health risk scores decreased in intervention group compared to questionnaire only group (p<0.001).  Exercise minutes per week and exercise programme attendance significant difference baseline to 24 months (p<0.05).  Between group differences reported for 12 months only, not reported at 24 months. |
| **Pasalich et al. 2013**  **Country:** Australia  **Study design:** Follow up survey as part of cohort study  **Length of follow up:** 6 months | **Number of participants: n=349**  **Age:** mean 65 60-70 years old  **Retirement:** 42% still employed  **Gender:** 50% male  **Education:** :95% high school and above  **Ethnicity:** NR **Other inclusion/ exclusion criteria:** Insufficiently active older adults | **Intervention content**  Physical Activity and Nutrition for Seniors programme. 6 month home-based intervention. Received a booklet containing information on recommended physical activity levels, dietary guidelines and encouraged goal setting. Also charts, newsletters, pedometers provided. Contacted via telephone or email to offer additional support.  **Control condition if applicable:** No intervention  **Data collection methods:** International Physical Activity Questionnaire  **Outcome measures:** Days per week and minutes of walking and moderate intensity activity | T tests indicated significant improvement for the intervention group for strength activity compared to little change in control group (p>0.1) at post-programme. At follow up the control group had declined, while intervention group remained above baseline level, although no significant difference. No difference at follow up for controls or intervention group, intervention group had significantly declined from immediate post programme. Outcomes relating to diet were more positive. |
| **Walker et al. 2009**  **Country:** USA  **Study design:** RCT  **Length of follow up:** 12 months | **Number of participants:** n=225  **Age:** 50-69, mean age 57  **Retirement:** 46% employed full time, 18% employed part time, 33% not employed  **Gender:** Female  **Education:** 70% some college or higher, around 45% income above 40k.  **Ethnicity:** 95% white **Other inclusion/ exclusion criteria:** English-speaking; able to use a computer with assistance to complete a survey; access to a video recorder to view physical activity demonstration tapes; able to answer no to all questions on the Physical Activity Readiness Questionnaire. Not being in the maintenance stage for all 3 components of readiness for change in physical activity | **Intervention content**  Wellness for Women study. Women received by mail either 18 generic newsletters or 18 newsletters computer-tailored. Feedback on data collected provided every month. Pedometers provided.  **Control condition if applicable:** Two intervention arms only  **Data collection methods:** Questionnaires, physical measures  **Outcome measures:** : 7 day recall questionnaire, cardiorespiratory fitness, body fat, blood pressure, Exercise Benefits/Barriers Scale, Healthy Eating Benefits/Barriers Scales, Self-Efficacy for Exercise Habits Scale; Self-Efficacy for Eating Habits Scale, Family Support for Exercise Habits Scale, Friend Support for Exercise Habits Scale, Family Support for Healthy Eating Habits Scale, Friend Support for Healthy Eating Habits Scale | **Main findings**  All behavioural and biomarkers of physical activity had significant main effects for time over the intervention period. Both groups improved significantly from baseline to 6 months on all measures. There were no significant differences between the newsletter groups in achievement of any target at 6 months. At 12 months, a significantly higher proportion of the tailored newsletter group had achieved the activity behavioural outcome target of at least 210 minutes of moderate or greater intensity activity weekly (30 minutes daily on 7 days each week). |
| **Walker et al. 2010**  **Country**: USA  **Study design:** RCT  **Length of follow up:** 24 months (12 month intervention plus 12 month follow up) | **Number of participants: n=**225  **Age**: 50-69  **Retirement:** As above  **Gender:** Female  **Education**: As above  **Ethnicity**: As above **Other inclusion/ exclusion criteria:** As above | **Intervention content**  As above  **Control condition if applicable:** As above  **Data collection methods**:  **Outcome measures**: As above | **Main findings**  From 12 months to 24 months, both groups maintained levels of moderate or greater activity, stretching exercise, and flexibility, but declined in cardiorespiratory fitness; only the tailored newsletter group maintained levels of strength exercise and lower body strength, while the generic newsletter group showed a decrease in strength exercise and lower body strength.  For the entire period from 12 to 24 months, neither time by group interaction was significant, but the main effect for time was significant for the outcome of at least 210 minutes of weekly moderate or greater activity. |

**Home-based interventions providing a pedometer/accelerometer**

| **Finkelstein et al. 2008**  **Country**: USA  **Study design:** RCT  **Length of follow up:** immediate | **Number of participants:** N= 70  **Age**: mean 59.4 intervention and 61.2 control, min 50 max 85  **Retirement**: 28.6% intervention and 43.3% control retired and not working  **Gender:** 23.8%/26.7% male  **Education**: 85%/70% college degree or higher, 40% household income greater than $50.000  **Ethnicity**: non-white 4.8% intervention 6.7% control **Other inclusion/ exclusion criteria:** at least aged 50, less than 2 hour exercise per week | **Intervention content**  Attended initial meeting, wore pedometer for 4 weeks, given $50 initial payment and subsequent payments dependant on number of aerobic minutes per day  **Control condition if applicable:** Participants attended an initial meeting and wore the pedometer, given $75 payment for taking part, wearing the pedometer daily and returning all study materials.  **Data collection methods**: Pedometer and diary  **Outcome measures:** Aerobic minutes as measured by a pedometer and self-report log | **Main findings**  Adjusted treatment effect - just over 16 more aerobic minutes in intervention group (p>0.001). Small difference between full time employees and retirees (retired more minutes) but this was not significant.  Control group average 20 minutes per day, slight decrease over the 4 weeks. Intervention group average 35 minutes per day (1.8 hours per week more than controls) with little change over the 4 weeks. |
| --- | --- | --- | --- |
| **Koizumi et al. 2009**  **Country**: Japan  **Study design:** RCT  **Length of follow up:** Immediate | **Number of participants:** N= 68  **Age**: mean 67 (SD 4)  **Retirement**: NR  **Gender:** All female  **Education**: NR  **Ethnicity**: NR **Other inclusion/ exclusion criteria:** Excluded if any gait abnormality,or taking medication. | **Intervention content**  Lifestyle physical activity (LIFE) – accelerometer given to all participants. Recommended participants accumulated 9000 steps and 30 minutes of moderate intensity physical activity per day, data provided to participants for previous 2 weeks with recommendations made. Intervention over 12 weeks.  **Control condition if applicable:** Wore same device but was locked and collected no data  **Data collection methods**: Accelerometer recorded data  **Outcome measures:** Number of daily steps, moderate intensity activity, 12 minute walk test | **Main findings**  First 2 weeks used as baseline data outcomes a combining of 11^th^ and 12^th^ week. The intervention group increased steps average by 16% (7811 to 2620 p<0.01). Moderate intensity activity also increased by 53% (17.83 to 27.23 p<0.01). No change in control group. Walk time test also improved for intervention group by 10% (p<0.01). |
| **Sawchuck et al. 2008**  **Country**: USA  **Study design:** RCT  **Length of follow up:** immediate | **Number of participants:** N= 125  **Age**:50-74, average age 58 years  **Retirement**: 27% intervention group 1 employed 16% group 2  **Gender: 74% female**  **Education**: NR  **Ethnicity**: American Indian **Other inclusion/ exclusion criteria:** having a sedentary lifestyle, living within 2 hours of study site | **Intervention content**  Two 6 week interventions. Both groups received baseline and final clinic visit with two ten minute phone calls in week 2 and 4. Pedometer intervention group given instructions and trained in the use of a pedometer.  Activity monitoring only interventiion group given daily monitoring sheets to complete and suggested activities and exercises and an educational hand out on health benefits of exercise.  **Control condition if applicable:** Two intervention arms  **Data collection methods**: Questionnaires, walk test, pedometer data, self-report booklet  **Outcome measures:** CHAMPS questionnaire, SF-36, 6 minute walk test, total daily step counts, activity log | **Main findings**  The frequency of walking increased for both groups from baseline (p<0.01). The addition of the pedometer therefore did not lead to appreciable differences.  No difference between groups on CHAMPS, SF-36, BMI-adjusted mean distance travelled during the 6 min walk. Combined results for the two groups showed improved calorific expenditure for all exercise-related activities (p<0.001) and frequency of moderate-intensity exercise related activities (p<0.001). |
| **Strath et al. 2011**  **Country**: USA  **Study design:** pilot RCT  **Length of follow up**: immediate | **Number of participants:** N= 81  **Age**: 55-80 years, mean 63.8 (SD 6)  **Retirement**: NR  **Gender:** 83% female  **Education**: majority had completed a college degree (73%) and 65% earned more than $35,000  **Ethnicity**: NR **Other inclusion/ exclusion criteria:** self-report engaging in activity less than 30 minutes per day 5 days a week, wore pedometer to test less than 7500 steps per day | **Intervention content**  Four intervention arms all 12 weeks – standard education consisting of educational physical activity material bi-weekly by email with 6 mailings received. Secondly, standard education + pedometer and pedometer log in mail with instructions on how to use and log daily steps with envelopes to mail logs back on a weekly basis. Thirdly, pedometer + 6 individualised 2 page education booklet based on stages of change motivational literature including perceived barriers and effective strategies to overcome these. Fourth intervention – pedometer + individual education + telephone call bi-weekly (6 telephone calls of 10 minutes).  **Control condition if applicable:** Four intervention arms  **Data collection methods**: pedometer data, SF-36  **Outcome measures:** daily steps, health-related quality of life | **Main findings**  Third and fourth intervention group participants significantly greater number of daily steps than groups one and two (p<0.001) with no significant difference between these two groups (p=0.893).  Those increasing their daily steps to target threshold of 2000 were - group 2 44%, group 3 75%, group 4 79%.  25% of group 2, 56% of group 3 and 64% of group 4 increased to target threshold of 3000 steps per day. |

**Computer-based interventions**

| **Hageman et al. 2005**  **Country**: USA  **Study design:** RCT  **Length of follow up:** 3 months | **Number of participants:** N= 31  **Age**:50-69 Mean 56.1  **Retirement**: 6.7% retired, 73% full time and 6.7% part time employed  **Gender:** all female  **Education**: 50% college graduate or higher  **Ethnicity**: 86% White **Other inclusion/ exclusion criteria:** English speaking, answered no to all items on Physical Activity Readiness Questionnaire, less than 30 mins physical activity on 5 or more days per week, have access to computer and internet in home | **Intervention content**  3 newsletters delivered via the internet at baseline, one months and two months. These were individually tailored to participants based on baseline assessments or standard.  **Control condition if applicable:** Standard newsletter  **Data collection methods**: Questionnaire, physical measures  **Outcome measures:** Modified 7 day activity recall questionnaire, Rockport Walking Fitness Test, body composition, height, weight, flexibility, Benefits and Barriers scales, Self-efficacy for Exercise Habits Scale | **Main findings**  No significant change for any self-reported activity or perceived benefits of activity. Both groups declined in regard to perceived barriers (p=0.025). Self-efficacy increased for standard group but decreased for tailored group (p=0.510 and p=0.018). |
| --- | --- | --- | --- |
| **Irvine et al. 2013**  **Country**: USA  **Study design:** RCT  **Length of follow up:** 6 months (assessed at pre-test, 12 weeks, and 6 months). | **Number of participants:** 368 sedentary men and women (subgroup analysis, original trial n=878)  **Age**: M=60.3; SD 4.9)  **Retirement:** NR  **Gender:** 69% female  **Education**: 82% had at least some college education  **Ethnicity**: 59% Caucasian **Other inclusion/ exclusion criteria:** NR | **Intervention content if applicable**: Active After 55, a multiple-visit Internet program to enhance functional ability, mobility, and physical activity of older adults. Uses automated video and text support and education, with the option to change or increase their exercise plan.  **Control condition if applicable:** Control group did not have access to the intervention.  **Data collection methods**: 14 Self-rated measures (questionnaires including SF12)  **Outcome measures:** Frequency and duration of intentional physical activities, fitness level, activity goals, and barriers to exercise | **Main findings**  Significant treatment effects at post-test (p=0.001; large effect size) and at 6 months (p=0.001; medium effect size).  At post-test, intervention participation showed significant improvement on 13 of 14 outcome measures compared to the control participants.  At 6 months, treatment participants maintained large gains compared to the control participants on all outcome measures. The multivariate model at follow-up was significant in which the Tx participants were found to maintain large gains compared to the Ctrl participants, F (14, 337) = 3.08, p<0.001, eta-square = 0.11. |
| **Peels et al. 2012**  **Country**: Netherlands  **Study design:** Process evaluation  **Length of follow up:** NA | Describes development of intervention only | Describes development of Active Plus intervention only. Intervention evaluated in the Van Stralen et al./Peels et al. papers. | **Main findings**  Describes how the findings from the Active Plus intervention were used to develop a new web-based programme. Four intervention arms – basic print-delivered and web-delivered, printed and environmental information, and fourthly, web and environmental information. |
| **Peels et al. 2012**  **Country:** The Netherlands  **Study design:** cluster RCT  **Length of follow up:** 4 months | **Number of participants:** N=1248  **Age**: Over 50  Mean 61.6 to 64.1 (SD 7.2-8.3)  **Retirement**: NR  **Gender**: 43.7-51.8% male  **Education**: 41.5-49.5% low education  **Ethnicity**: NR **Other inclusion/ exclusion criteria:** NR | **Intervention content**  Active Plus intervention**.** Four intervention arms – basic print-delivered and web-delivered, printed and environmental information, and fourthly, web and environmental information.  Intervention aims to influence awareness, initiation, and maintenance of physical activity. Participants received advice on 3 occasions which was tailored to them based on previous responses. Advice included general benefits of physical activity, feedback based on their stage of change, advice regarding social support, practical possibilities and action plans. The additional environmental component consisted of local possibilities and initiatives in their neighbourhood.  **Control condition if applicable:** Waiting list  **Data collection methods**: questionnaire  **Outcome measures:** Dutch Short Questionnaire to Assess Health Enhancing Physical Activity | **Main findings**  The printed intervention achieved a higher participation rate than a web-based intervention (19% versus 12%)  Drop out significantly higher in web based intervention (53% versus 39% p<0.001)  No specific user characteristics were associated with drop out for different interventions, with low intention to be physically active predicting drop out for both delivery modes (p<0.001). |
| **Peels et al. 2013**  **Country**: Netherlands  **Study design:** cluster RCT  **Length of follow up:** 1 year | **Number of participants:** N=1248  **Age**: Over 50  Mean 61.6 to 64.1 (SD 7.2-8.3)  **Retirement**: NR  **Gender**: 43.7-51.8% male  **Education**: 41.5-49.5% low education  **Ethnicity**: NR **Other inclusion/ exclusion criteria:** NR | **Intervention content**  Active Plus (see other Peels/Van Stralen papers)  **Control condition if applicable:** Waiting list  **Data collection methods**: questionnaire  **Outcome measures:** Dutch Short Questionnaire to Assess Health Enhancing Physical Activity | **Main findings**  At 12 months the four intervention conditions as a whole were effective in increasing weekly days of sufficient physical activity (p=0.005 ES 0.18) but ineffective in increasing weekly minutes of physical activity (p=0.071 ES 0.20). |
| **Van Stralen et al. 2009**  **Country**: Netherlands  **Study design:** RCT  **Length of follow up:** 6 months | N = 1971  **Age**: average 64 years old (SD= 8.6)  **Retirement:** 47% employed.  **Gender:** NR  **Education**: 48% low level of education  **Ethnicity**: NR **Other inclusion/ exclusion criteria:** average BMI of 25.5 | **Intervention content**  Computer-tailored letters delivered over 4 months:  (a) basic computer-tailored print intervention with personalized physical activity advice.  (b) plus environmentally computer-tailored print intervention additionally received environmentally focused computer-tailored information about physical activity opportunities in their neighbourhood combined with access to a forum and e-buddy system on a website.  **Control condition if applicable:** No-intervention  **Data collection methods**: Questionnaires  **Outcome measures:** Dutch Short Questionnaire to Assess Health Enhancing Physical Activity, scale to evaluate process | **Main findings**  At 6 months both intervention groups had enhanced awareness of their activity levels compared to controls (p<0.001 OR 1.67 intervention OR 1.64 intervention plus) with no significant difference between the intervention types. |
| **Van Stralen et al. 2009**  **Country**: Netherlands  **Study design:** Further analysis of data  **Length of follow up:** 6 months | N = 1971  **Age**: average 64 years old (SD= 8.6)  **Retirement:** 47% employed.  **Gender:** NR  **Education**: 48% low level of education  **Ethnicity**: NR **Other inclusion/ exclusion criteria:** average BMI of 25.5 | **Intervention content**  See above  **Control condition if applicable:** No-intervention  **Data collection methods**: Questionnaires  **Outcome measures:** Total weekly days and total weekly minutes of physical activity (self-reported) | **Main findings**  The intervention plus condition had a significant effect on cycling compared to basic intervention (p<0.01).  Intervention plus condition had significant effect on perceived physical activity possibilities (more than both controls and basic intervention p<0.05). |
| **Van Stralen et al. 2010**  **Country**: Netherlands  **Study design:** Cluster RCT  **Length of follow up:** 12 months | **Number of participants:**  N = 1971  **Age**: average 64 years old (SD= 8.6)  **Retirement:** 47% employed.  **Gender:** NR  **Education**: 48% low level of education  **Ethnicity**: NR **Other inclusion/ exclusion criteria:** average BMI of 25.5 | **Intervention content**  See above  **Control condition if applicable:** No-intervention  **Data collection methods**: Questionnaires  **Outcome measures:** Total weekly days and total weekly minutes of physical activity (self-reported) | **Main findings**  A significant intervention effect was found for the environmentally tailored intervention - increased total weekly minutes of physical activity by one hour per week more (BI environment = 62.0; 95% CI=7.4–116.6; p<0.05) compared with the control condition.  No significant intervention effects were found for the basic intervention condition compared with the control group.  Participants in the environmentally tailored intervention increased their physical activity behaviour by almost 50 minutes per week more than participants in the basic intervention (BI environment vs: I basic =48.5; 95% CI=–6.3–103.3; p=0.08). |
| **Van Stralen et al. 2011**  **Country**: Netherlands  **Study design:** cluster RCT  **Length of follow up:** 6 months | **Number of participants:** randomisation at the level of Municipal Health Councils N=6. N=2000 participants  **Age**: over 50 mean 64 (SD 8.6)  **Retirement**: 47% employed  **Gender:** 57% female  **Education**: 48% low level of education (baseline differences for this factor between groups)  **Ethnicity**: NR **Other inclusion/ exclusion criteria:** councils with at least 20,000 inhabitants | **Intervention content**  See above  **Control condition if applicable:** Waiting list  **Data collection methods**: Questionnaires  **Outcome measures:** Dutch short questionnaire to assess health-enhancing physical activity measured self-report days and minutes of physical activity, also potential mediators – awareness, attitude, social influences, motivation, self-efficacy, intention, commitment, perceived environment, strategic planning, action planning and coping planning | **Main findings**  Both groups increased total weekly days of physical activity from 2.3 (SD 2.2) to 4.7 (SD 2.0). Control had also slightly increased (from 4.0 to 4.3). Significant effects for both intervention groups (p<0.01) compared to controls. Small ES (0.18 for both) with no difference between effect of the different conditions for total weekly days. Only the plus intervention had a significant effect on total minutes of physical activity per week compared to controls (ES 0.19). |
| **Wijsman et al. 2013**  **Country**: Netherlands  **Study design:** RCT  **Length of follow up:** post intervention | **Number of participants:** N= 235  **Age**:60-70 mean 64.7 intervention group (SD 3) 64.9 control (SD 2.8)  **Retirement**: NR  **Gender:**39.5%/42.2% female  **Education**: 55.5% high level intervention group, 57.8 controls. Low level 5.9%/1.7%.  **Ethnicity**: NR **Other inclusion/ exclusion criteria:** no history of diabetes, absence of disability, usage of a computer with internet, less than 3 hours of walking/cycling per week | **Intervention content**  Commercially available web based programme. Three elements – accelerometer monitor worn continuously, personal website, personal e-coach. After 8 days of measuring participants given individual goals with data used for feedback and email coaching and advice.  **Control condition if applicable:** Waiting list  **Data collection methods**: Questionnaire, accelerometer data, body measurement  **Outcome measures:** total activity count, body weight, body fat | **Main findings**  Intervention group after 13 weeks daily physical activity as measured by the ankle accelerometer increased by 46% (p<0.001) compared to control group increase of 12% (p<0.001). There was a small increase in moderate-vigorous activity in the intervention group (mean increase 11.1 minutes per day) compared to mean decrease in control group (p=0.001 relative difference). |

**Community-wide initiatives**

| **Ackermann et al. 2005**  **Country**: USA  **Study design:** Cluster RCT  **Length of follow up:** 4 months | **Number of participants** 31 physicians and nurse practitioners.  336 patients.  **Age**: patients aged 50 and older (mean age 66).  **Retirement:** NR  **Gender:** 99% Male  **Education**: NR  **Ethnicity**: NR  **Other inclusion/ exclusion criteria:** 63% not regularly exercising. | **Intervention aims and content**: Primary care providers (PCP) were trained to offer referrals to community exercise programs for patients who reported before their clinic visit that they were “contemplative’ about regular exercise.  **Control condition if applicable:** randomized to a physical activity counselling intervention or control condition (counselling about tobacco cessation).  **Data collection methods**: Interviews – physical activityCE questionnaire (Physician-based Assessment and Counselling for Exercise).  **Outcome measures:** exercise stage-of-change, proportion of participants reporting regular physical activity. | **Main findings**  Forty-five percent of all intervention patients and 35% of controls reported receiving exercise advice (p=0.07). Intervention patients who were contemplative about exercise were even more likely to receive exercise advice than contemplative controls (59% vs 38%; p=0.02). After 4 months, 35% of all intervention patients reported regular exercise, compared with 28% of controls (p=0.06).  Primary providers are more likely to offer exercise advice when informed whether patients are contemplative about exercise. |
| --- | --- | --- | --- |
| **Coronini-Cronberg et al. 2012**  **Country**: UK  **Study design:** Before/After (repeated cross sectional survey)  **Length of follow up:** NA | **Number of participants:** n=16911  **Age**: 60 years and older (eligible for bus pass)  **Retirement:** NR  **Gender:** NR  **Education**: NR  **Ethnicity**: NR  **Other inclusion/ exclusion criteria:** none | **Intervention aims and content** Introduction of free bus pass to over 60s.  **Control condition if applicable:** NA  **Data collection methods**: 2005–2008 National Travel Survey. Interview, and a 1-week travel diary.  **Outcome measures:** active travel (walking, cycling, and use of public transport), use of buses, and walking 3 or more times per week. | **Main findings**  Having a free pass was significantly associated with greater active travel among both disadvantaged (adjusted odds ratio [AOR]=4.06; 95% confidence interval [CI =3.35,4.86; p<0.001) and advantaged groups (AOR = 4.72; 95%CI=3.99, 5.59; p<0.001); greater bus use in both disadvantaged and advantaged groups (AOR=7.03; 95%CI=5.53, 8.94; p<0.001 and AOR=7.11; 95% CI = 5.65, 8.94; p<0.001, respectively); and greater likelihood of walking more frequently in the whole cohort (AOR=1.15; 95%CI = 1.07, 1.12; p<0.001). |
| **Kamada et al. 2013**  **Country**: Japan  **Study design:** Cluster RCT  **Length of follow up:** 1 year | **Number of participants:** residents targeted in 12 communities each with median population of 1292 (15,504?), 4414 provided evaluation data  **Age**:40-79 sample providing data mean age 60/61 (SD 10)  **Retirement**: 64-71% employed  **Gender:** 44-47% male  **Education**: mean 11 years  **Ethnicity**: NR  **Other inclusion/ exclusion criteria:** all communities within a region (32) 12 of these randomly selected stratifying for population density (in each intervention group one high, one moderate, one low population density). For survey those in long term care, or unable to walk unaided excluded. | **Intervention aims and content** Community wide campaign to promote exercise (COMMUNICATE). Three intervention arms – mainly walking behaviour (aerobic activity), mainly stretching exercises (flexibility and muscle strengthening), and the third group all activities promoted.  **Control condition if applicable:** 9 communities in intervention, 3 in control condition. Control communities had no intervention.  **Data collection methods**: Baseline and post intervention survey  **Outcome measures:** number engaging in regular physical activity, daily and weekly reported walking time, frequency of flexibility activity**.** | **Main findings**  Baseline control group 64.6% of sample reported engaging in regular physical activity, intervention group 63.9%. Post campaign control group decreased slightly to 60.3%, intervention group also decreased to 58.7%. |

**Table summarising previous reviews**

|  | **Included studies** | **Included population & intervention** | **Summary findings** |
| --- | --- | --- | --- |
| Allender et al. 2008 [1] | 3 papers related to change in employment status (none retirement), any design | All ages “life change events” and participation in PA.  All papers Australian women from same dataset, associational data only, 22-27 year olds changing employment. | No relevant findings |
| Barnett et al. 2012 [2] | 19 studies longitudinal or cross sectional | Studies comparing retired to not retired or  longitudinal studies across retirement transition (no age)  Associations and predictors of PA at retirement transition. | Associational data only.  Exercise and leisure time PA increase in retirement but whether total PA increases is unclear.  Low SES associated with decrease in PA and higher with increase. |
| Barnett et al. 2012 [3] | 5 qualitative studies | Studies referred to transition to retirement | Themes – concepts of PA, motives for and challenges to PA in retirement.  Compared to findings from other review – factors influencing PA may be: expected health benefits; lifelong PA patterns; opportunities for socialising and personal challenges; desire for new routine; lack of time; low perceived value of recreational PA |
| Chase 2013 [4] | 20 studies | 60 and over  PA interventions | Cognitive only and combined cognitive-behavioural interventions more successful in changing behaviour, however behavioural only interventions better long term change in behaviour. Self-efficacy common construct in interventions. |
| Conn et al. 2002 [5] | 43 studies with control group | Over 60s  PA interventions | ES 0.26 (CI 0.05). Larger ESs for patient populations, interventions without health education, focusing specifically on activity, less than 90 days FU |
| Conn et al. 2003 [6] | 17 RCTs | Over 65s  PA interventions | 7/17 findings not significant, unable to identify components or attributes linked to outcomes |
| Engberg et al. 2012 [7] | 11 studies related to change in employment status (7 retirement) , longitudinal/cross sectional | All ages  Life events and changes in leisure PA | Associational data – retirement associated with increase in PA in 6 studies and decline in PA in one. One study women less increase in PA following retirement than men (31 mins per week vs 42 mins per week), one study both genders more likely to engage in sports activities in groups than alone, two studies conflicting evidence whether physical demands at work prior impacted on PA change after retirement. |
| Frost et al. 2010 [8] | 15 reviews and 1 review of reviews of exercise and activity (only 5 of direct relevance). | Age 50+ no specific disease or problems  Interventions in primary care and community for health and wellbeing in later life.  Exercise interventions encompassed flexibility, resistance training, aerobic, balance. | Older people encounter barriers initiating and adhering to exercise programmes such as lack of confidence to exercise and a belief that exercise is likely to do more harm than good.  Whilst older people can be encouraged to exercise, the  evidence tends to be derived from white, well-educated populations which don’t include those who are at greatest risk of functional decline.  Gains dependent on long term adherence and exercise behaviours generally established earlier in life and are hard to change.  Most promising interventions offer written reminders, are tailored to individuals and generate feelings of enjoyment and satisfaction.  Important to make an impact “upstream” before retirement. |
| Hobbs et al. 2013 [9] | 32 papers (21 studies) RCTs only | Adults aged 55-70  Behavioural  PA interventions | Interventions led to PA improvement at up to 12 months (pooled ES 0.19), unclear beyond this.  No dose-response effect, mode of delivery not important but individual tailoring may be, also type of intervention (walking/exercise classes) |
| King et al. 1998 [10] | 29 studies with comparator group | Over 50s  PA interventions | Highlights gaps in evidence rather than evaluating effectiveness |
| Muller & Khoo 2014 [11] | 17 papers (16 studies RCT/quasi experimental) | Over 50s  Non-face-to-face interventions for PA | 14 of 17 reported significant increase in PA. Eight of 9 reporting longer term FU found effect maintained. All studies Social Cognitive Theory or Trans-theoretical Model of Behaviour Change. |
| Taylor et al. 2004 [12] | Descriptive overview only | Older adults  PA interventions | Highlights importance of PA for wellbeing, mentions elderly particularly vulnerable to inequalities. |
| Van der Biij et al. 2002 [13] | 38 RCTs | Average age 50 or over (at least age 40) no diagnosed disease  PA interventions | Group based and educational interventions effective for PA in the short term. Limited data for home based interventions. Effect for educational however faded at longer FU, data insufficient to make a judgement on effectiveness of group based in longer term. No evidence for behavioural reinforcement strategies (e.g. reminders) on effectiveness. Participation rates comparable group and home based. Group based higher participation than home based in long term however. |
| Von Bonsdorff & Rantanen 2011 [14] | 16 studies (only 2 described PA outcomes)  Experimental or longitudinal design | 60 or over  Voluntary work | The 2 papers reported activity levels increased |
| Wilson & Palha 2007 [15] | 20 papers any design | Health promotion immediately before or following retirement. Papers which differentiated findings by age or life stage. | Associations and predictors data. Age differences in use of activity or sports-related services (more use 65-74) than older. One study - increase in activity at retirement for both sedentary and active workers. Work or jobs taken after retirement a barrier to PA. Positive self-perceptions of ageing predicted preventive health behaviours such as PA.  PA of all types declined in men over time, recreational activity increased when first retired then levelled off before declining after 70. PA of all types declined after 63 for women. Men more time for PA than women after retirement and recreational PA more socially acceptable among retired men. Accessibility and race barriers. Those participating in wellness programme before retirement more likely to participate after. |

1. Allender S, Hutchinson L, Foster C. Life-change events and participation in physical activity: a systematic review. Health Promot Int 2008; 23: 160-72 doi: 10.1093/heapro/dan012.

2. Barnett I, Ogilvie D, Guell C. Physical activity and transitioning to retirement: a systematic review. Am J Prev Med 2012; 43:329-336.

3. Barnett I, Guell C, Ogilvie, D. The experience of physical activity and the transition to retirement: a systematic review and integrative synthesis of qualitative and quantitative evidence. Int J Behav Nutr Phys Act 2012; 9:97 doi: 10.1186/1479-5868-9-97.

4. Chase JA. Physical activity interventions among older adults: a literature review. Res Theory Nurs Pract 2013; 27: 53-80.

5. Conn VS, Minor MA, Burks KJ, Rantz MJ, Pomeroy SH: Integrative review of physical activity intervention research with aging adults. J Am Geriatr Soc 2003, 51:1159-1168.

6. Conn VS, Valentine JC, Cooper HM: Interventions to increase physical activity among aging adults: a meta-analysis. Ann Behav Med 2002, 2:190-200.

7. Engberg E, Alen M, Kukkonen-Harjula K, Peltonen JE, Tikkanen HO, Pekkarinen H. Life events and change in leisure time physical activity: a systematic review. Sports Med 2012; 42: 433-447.

8. Frost H, Haw S, Frank J. Promoting Health and Wellbeing in Later Life: Interventions in Primary Care and Community Settings. Edinburgh: Scottish Collaboration for Public Health Research and Policy, 2010.

9. Hobbs N, Godfrey A, Lara J, Errington L, Meyer TD, Rochester L et al. Are behavioral interventions effective in increasing physical activity at 12 to 36 months in adults aged 55 to 70 years? A systematic review and meta-analysis. BMC Medicine 2013, 11:75 doi:10.1186/1741-7015-11-75.

10. King AC, Rejeski WJ, Buchner DM. Physical activity interventions targeting older adults. A critical review and recommendations. Am J Prev Med 1998; 15: 316-333.

11. Muller AM, Khoo S. Non-face-to-face physical activity interventions in older adults: a systematic review. Inter J Behav Nutrition Phys Activ 2014, 11:35 doi:10.1186/1479-5868-11-35.

12. Taylor A, Cable N, Faulkner G, Hillsdon M, Narici M, Van Der Bij A. Physical activity and older adults: a review of health benefits and the effectiveness of interventions. J Sports Sci 2004; 22:703-725.

13. Van der Bij AK, Laurant MGH, Wensing M. Effectiveness of physical activity interventions for older adults - A review. Am J Prev Med 2002; 22(2):120-133.

14. Von Bonsdorff M, Rantanen T. Benefits of formal voluntary work among older people. A Review. Ageing Clin Exp Res 2011;23: 162-169.

15. Wilson DM, Palha P. A Systematic Review of Published Research Articles on Health Promotion at Retirement. J Nurs Scholar 2007; 4: 330-337 doi: 10.1111/j.1547-5069.2007.00189.x.

**Table detailing the outcomes measured in the included studies**

| **Objective measures** | **Activity**  Accelerometer  Pedometer (daily steps/aerobic minutes)  Retention and participation in programmes |
| --- | --- |
|  | **Fitness**  One mile walk time  12 minute walk test  12 minute swim  1.6 km walk time  Record of illness and injury  Health benefit company data on claims made  Rockport Walking Fitness Test |
|  | **Bio-physical**  Blood pressure  Body fat  Cardiovascular risk  Body composition  BMI  Weight  Waist circumference  Biotrainer data  Senior Fitness tests of muscle strength, endurance and balance |
| **Self-reported** | **Activity**  7 day activity recall questionnaire (or modified version)  Achievement of recommended minimum levels of moderate intensity physical activity  Community Activities Model Program for Seniors Questionnaire (CHAMPS) used to calculate calorific expenditure  Compendium of Physical Activity Tracking Guide  Dutch Short Questionnaire to Assess Health-Enhancing Physical Activity  Exercise history questionnaire  Exercise Habits Scale  Health habits questionnaire  Human Activity Profile  International Physical Activity Questionnaire -Short Form  Leisure Time Exercise Questionnaire  Maximum current activity  Measure of Older Adults’ Sendentary Time  Minutes of moderate to vigorous physical activity  National Travel Survey  Older adults sedentary behaviour  physical activity scale for the elderly  Paffenbarger sports and exercise index  Reported level of regular physical activity  Self-report daily log of activity  Sitting time (minutes per week)  Time spent walking  Total weekly days and total weekly minutes of physical activity  Travel diary |
|  | **Fitness**  Comparative fitness rating  Perceived fitness score |
|  | **Psychosocial correlates**  Barriers to Self-efficacy Scale  Benefits and Barriers scales  Exercise Motivation Scale.  Exercise self-efficacy Scale  Family Support for Exercise Habits Scale  Friend Support for  Physical Activity Group Environment questionnaire  Physical Activity Readiness Questionnaire  Physical improvement programme perceptions  Physician-based Assessment and Counselling for Exercise Questionnaire  Quality of Life scales (SF-36)  Reported awareness, attitude, social influences, motivation, intention, commitment, perceived environment, strategic planning, action planning and coping planning  Satisfaction with the intervention  Self-efficacy for Exercise Habits Scale  Social Support and Exercise Survey  Stage of Change Instrument |
|  | **Health correlates**  Behavioural Risk Factors Surveillance system  Center for Epidemiological Studies Depression Scale  Normative impairment index  Nottingham Health Profile questionnaire  Perceived Stress Scale  Satisfaction with body functioning  Self-reported physical performance, perceived functioning and well-being  Vitality Plus scale |

**Quality appraisal of the included studies**

**(Y=yes, potential bias present; N=no, no concerns regarding this type of bias)**

| Author/Year | 1.  *Selection bias* | 2.  *Performance bias* | 3.  *Attrition bias* | 4.  *Detection bias* | 5.  *Reporting bias* | Total  of  Nos |
| --- | --- | --- | --- | --- | --- | --- |
| Ackermann^27^ | Y | Unclear | N | Y | N | 2 |
| Armit^28^ | Unclear/not fully random | Unclear | N | Y | N | 2 |
| Burke^29^ | Unclear | Unclear | N | Y | N | 2 |
| Burman^30^ | Unclear | Unclear | Y | Y | N | 1 |
| Caperchione^31^ | Y | Y | N | Y | N | 2 |
| Castro^32^ | Unclear | N | Y | Y | N | 2 |
| Coronini-Cronberg^33^ | N | N | Y | Y | N | 3 |
| Costanzo^34^ | Y | N | Y | Y | Y | 1 |
| Cox^35^ | Y | N | Y | Y | N | 2 |
| Croteau^36^ | Y | N | Y | N | N | 3 |
| De Jong^37^ | Unclear | Unclear | Y | N | Y | 1 |
| De Jong^38^ | Unclear | Unclear | Y | N | Y | 1 |
| Dorgo^39^ | Unclear | Unclear | N | Unclear | Y | 1 |
| Elley^40^ | Y | N | N | N | N | 4 |
| Finkelstein^41^ | Y | N | N | N | N | 4 |
| Fries^42^ | Y | N | Y | N | N | 3 |
| Fries^43^ | Y | N | Y | N | N | 3 |
| Fujita^44^ | Y | N | N | Y | N | 3 |
| Goldstein^45^ | Y | N | N | Y | N | 3 |
| Hageman^46^ | Y | N | N | N | N | 4 |
| Halbert^47^ | Unclear | N | N | N | N | 4 |
| Hamdorf^48^ | Y | Unclear | Y | Y | N | 1 |
| Hamdorf^49^ | Y | Unclear | N | N | N | 3 |
| Hekler^50^ | Y | Y | N | N | N | 3 |
| Hooker^51^ | Y | Y | N | Y | N | 2 |
| Hughes^52^ | Y | N | Y | N | N | 3 |
| Irvine^53^ | Y | N | Y | N | N | 3 |
| Kamada^54^ | N | N | N | Y | N | 4 |
| King^55^ | Y | N | N | N | N | 4 |
| King^56^ | N | N | N | N | N | 5 |
| King^57^ | Unclear | N | N | Y | N | 2 |
| Koizumi^58^ | Unclear | N | Unclear | N | N | 3 |
| Lawton^59^ | Y | N | N | N | N | 4 |
| Marcus^60^ | Y | Y | Y | Y | Y | 0 |
| Martinson^61^ | N | N | N | Y | N | 4 |
| Martinson^62^ | N | N | N | Y | N | 4 |
| Opdenacker^63^ | Y | Y | Y | N | N | 2 |
| Opendacker^64^ | Y | Y | Y | N | N | 2 |
| Pasalich^65^ | Y | N | N | Y | N | 4 |
| Peels^66^ | Unclear | Unclear | NA | NA | N | 1 |
| Peels^67^ | Unclear | Unclear | NA | NA | N | 1 |
| Peels^68^ | Unclear | Unclear | Y | Y | N | 1 |
| Pereira^69^ | Unclear | N | N | Y | N | 3 |
| Petrella^70^ | Unclear | Unclear | N | N | N | 3 |
| Pinto^71^ | Y | Unclear | N | N | N | 3 |
| Prabu^72^ | Y | N | Y | N | N | 2 |
| Purath^73^ | N | N | Y | N | N | 4 |
| Rowland^74^ | Y | N | N | Y | N | 3 |
| Sawchuck^75^ | Y | Unclear | N | Y | N | 2 |
| Stevens^76^ | Y | Unclear | Y | Y | N | 1 |
| Strath^77^ | Y | N | N | N | N | 4 |
| Van Keulen^78^ | N | N | Y | Y | N | 3 |
| Van Stralen^79^ | N | N | N | Y | N | 4 |
| Van Stralen^80^ | N | N | Y | Y | N | 3 |
| Van Stralen^81^ | N | N | Y | Y | N | 3 |
| Van Stralen^82^ | N | N | Y | Y | N | 3 |
| Walker^83^ | N | N | N | Y | N | 4 |
| Walker^84^ | N | N | N | N | N | 5 |
| Werkman^85^ | N | N | N | N | N | 5 |
| Wijsman^86^ | Y | N | N | Y | N | 3 |
| Wilcox^87^ | Unclear | Y | Y | Y | N | 1 |
| Wilcox^88^ | Unclear | Y | Y | Y | N | 1 |
| Wilcox^89^ | Unclear | Y | Y | Y | N | 1 |
| Wilcox^90^ | NA | NA | Y | NA | NA | NA |
| Total of Nos | 13 | 38 | 33 | 26 | 59 |  |
